# *N*-methyl mesoporphyrin IX as a highly selective light-up probe for G-quadruplex DNA

**DOI:** 10.1142/s1088424619300179

**Published:** 2019-12

**Authors:** Ariana Yett, Linda Yingqi Lin, Dana Beseiso, Joanne Miao, Liliya A. Yatsunyk

**Affiliations:** Swarthmore College, Department of Chemistry and Biochemistry, 500 College Ave, Swarthmore, PA 19081, USA

**Keywords:** G-quadruplex DNA, *N*-methyl mesoporphyrin IX, light switch, fluorescence, selectivity, parallel G-quadruplex

## Abstract

*N*-methyl mesoporphyrin IX (NMM) is a water-soluble, non-symmetric porphyrin with excellent optical properties and unparalleled selectivity for G-quadruplex (GQ) DNA. G-quadruplexes are non-canonical DNA structures formed by guanine-rich sequences. They are implicated in genomic stability, longevity, and cancer. The ability of NMM to selectively recognize GQ structures makes it a valuable scaffold for designing novel GQ binders. In this review, we survey the literature describing the GQ-binding properties of NMM as well as its wide utility in chemistry and biology. We start with the discovery of the GQ-binding properties of NMM and the development of NMM-binding aptamers. We then discuss the optical properties of NMM, focusing on the light-switch effect — high fluorescence of NMM induced upon its binding to GQ DNA. Additionally, we examine the affinity and selectivity of NMM for GQs, as well as its ability to stabilize GQ structures and favor parallel GQ conformations. Furthermore, a portion of the review is dedicated to the applications of NMM-GQ complexes as biosensors for heavy metals, small molecules (*e.g.* ATP and pesticides), DNA, and proteins. Finally and importantly, we discuss the utility of NMM as a probe to investigate the roles of GQs in biological processes.

## Graphical Abstract

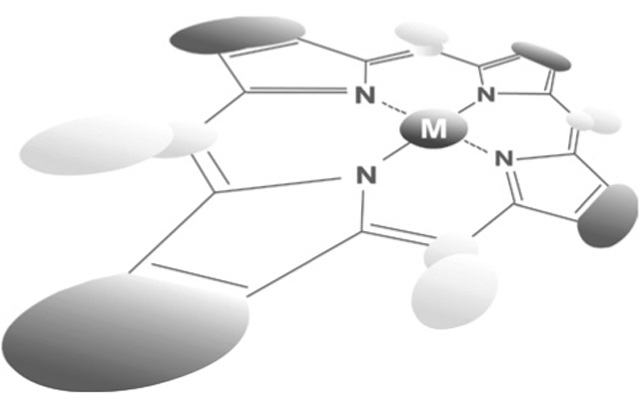

## INTRODUCTION

**Fig. 1.**
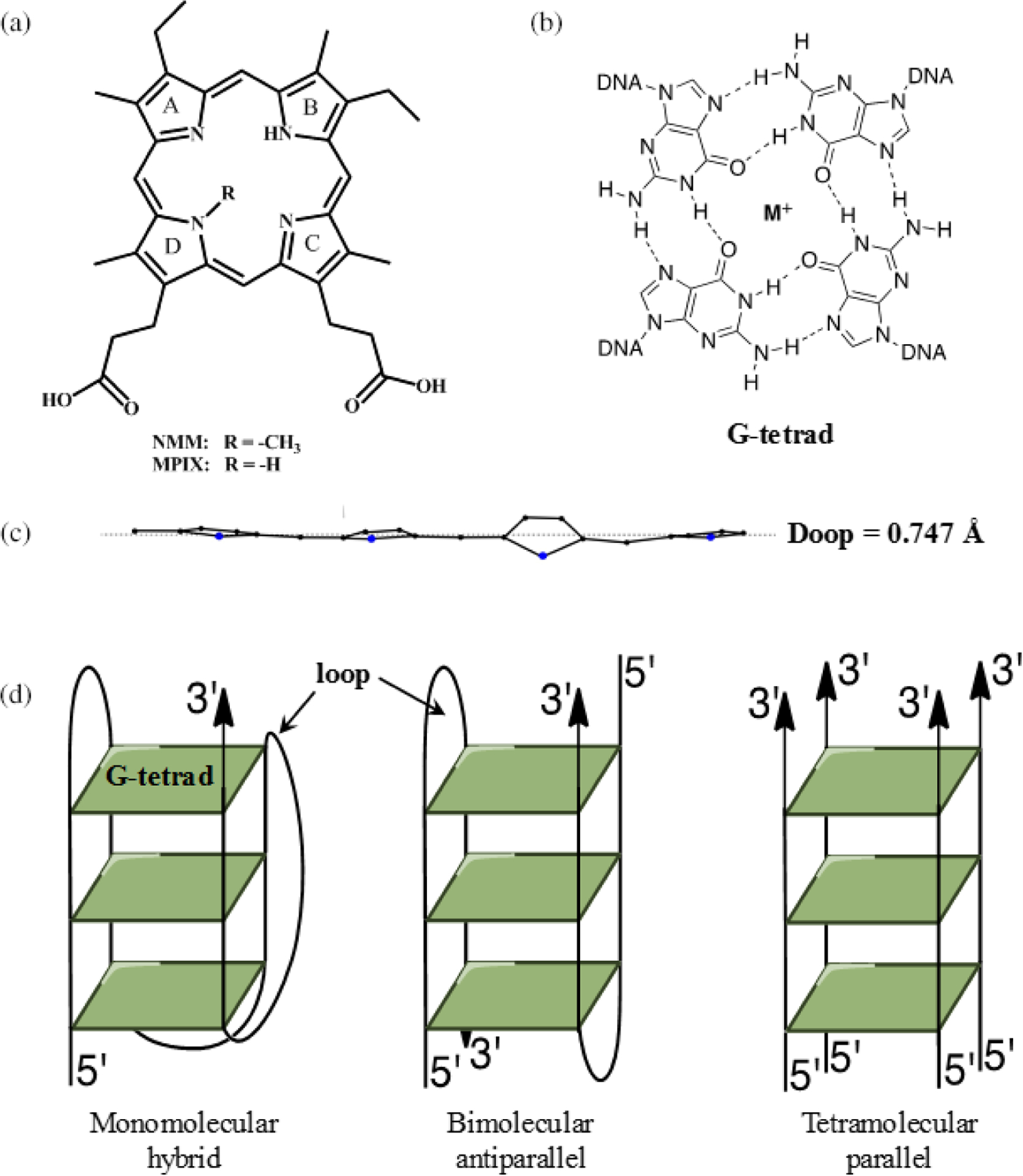
NMM, MPIX, and GQ DNA. (a) Structure of *N*-methyl mesoporphyrin IX (NMM) and *N*-methyl protoporphyrin IX (MPIX). (b) Structure of a G-tetrad. M^+^ is Na^+^ or K^+^. (c) Deviation of NMM from non-planarity. The data are taken from the NMM-Tel22 crystal structure, PDB ID 4FXM. The dotted line represents the mean plane. (d) Schematic representation of GQ topologies: hybrid, where three strands point up and one down (left); antiparallel with two strands pointing up and two strands pointing down (center); and parallel, where all four strands point in the same direction 5′ to 3′ (right)

*N*-methyl mesoporphyrin IX (NMM, Fig. 1a) is a non-symmetric, water-soluble porphyrin with excellent optical properties and exceptional selectivity for G-quadruplex (GQ) DNA. GQs are non-canonical DNA structures composed of two to four π–π stacked G-tetrads. Each G-tetrad is composed of four guanines connected *via* Hoogsteen hydrogen bonding and stabilized by a monovalent cation (Fig. 1b). Unlike double-stranded DNA (dsDNA), GQs display high structural diversity. They can be categorized by strand orientation into parallel, hybrid, and antiparallel geometries, as well as by the number of strands into mono-, bi-, and tetramolecular structures (Fig. 1d). This structural diversity makes GQs an interesting, yet challenging, topic for investigation. The importance of GQs stems from their involvement in a variety of biological processes. There is compelling evidence that GQs influence epigenetics, play protective roles at telomeres, define DNA replication origins in vertebrates, and serve as roadblocks during replication, transcription, and DNA repair, thereby linking them to cancer [1]. Therefore, small molecule ligands that modulate GQ structure and stability can be of great therapeutic value. In this review, we provide a detailed account of the GQ-binding properties and biological effects of the highly promising GQ ligand NMM.

### Chemical structure and physical properties of NMM

NMM (MW = 580 g/mol) is a commercially available compound that can be synthesized *via* methylation of mesoporphyrin IX (MPIX). It exists as a mixture of four regioisomers with the methyl group at one of the four core nitrogens (note, only one regioisomer is shown in Fig. 1a). In addition, each isomer exists as a racemic mixture of two enantiomers with the methyl group either up or down relative to the macrocycle ring. The calculated free energy barrier for enantiomer interconversion is 54.3 kcal/mol at 298 K, indicating that racemization would require temperatures in excess of 500 K to occur on a timescale shorter than decades [2]. The four regioisomers are difficult to separate. It is easier to separate them into two groups, with the methyl group on the pyrrole rings A–B and on the pyrrole rings C–D (Fig. 1a). The reported separation method starts with the methyl ester of NMM, and the separated compounds need to be hydrolyzed afterwards [3]. As far as we know, all work reported in the quadruplex field was done using a mixture of NMM isomers. Our laboratory attempted the separation following the protocol in [3] but did not succeed in obtaining pure regioisomers.

The presence of an *N*-methyl group on one of the pyrrole nitrogens leads to a large non-planar deviation in the porphyrin macrocycle [4]. This deviation, however, is highly localized on the pyrrole ring bearing the *N*-methyl group (Fig. 1c). Similar localization of distortion was reported for doubly methylated octaethyl porphyrin [4]. No experimental structure of NMM is available, but its geometry can be obtained from the reported crystal structures of NMM in complex with human telomeric DNA (AGGG(TTAGGG)_3_, named Tel22) [2] and with the *Bacillus subtilis* wild type [5] and mutant [6] ferrochelatases. In these structures, the overall deformation of the porphyrin macrocycle, expressed as Doop (deformation, out-of-plane), is 0.747, 1.581, and 1.839 Å, respectively. These numbers were calculated using the normal coordinate structural decomposition method, wherein the porphyrin plane was defined by the 24 core atoms, including nitrogens. These Doop values reveal that NMM distortion is highly dependent on its interacting partner. We have also estimated the geometry of NMM alone *via* energy minimization based on the NMM coordinates found in the Tel22-NMM complex and obtained a Doop of 1.068 Å [2]. The non-planarity of NMM has a strong effect on its physical properties as well as on its ability to interact with GQ DNA and proteins.

Another important feature of NMM is its two propionic acid side chains, which likely lead to its negative charge at physiological pH. As a charged non-planar molecule, NMM displays excellent solubility in water (in the mM range). Through serial dilution experiments, we have demonstrated that NMM does not aggregate in solution in the concentration range of 1–50 μM, which is used in the majority of chemical and biological studies [7]. Our data could also suggest that NMM exists as a stable dimer that does not dissociate or aggregate in this concentration range. NMM’s solubility contrasts with that of many other porphyrins, which are often only soluble in DMSO and display difficult-to-control aggregation phenomena. We typically work with aqueous, concentrated stocks of NMM (2–5 mM), which are stable for at least 6 months when kept at 4 °C in the dark. The water solubility and well-defined solution state of NMM, coupled with its excellent optical properties and selectivity toward GQ DNA (discussed below), makes NMM a highly suitable molecule for biological and biochemical studies.

### Discovery of NMM as a GQ ligand and the development of NMM aptamers *via* SELEX

NMM was first identified as a strong inhibitor of Fe^2+^ insertion into protoporphyrin IX (PPIX) in a reaction catalyzed by ferrochelatase, a terminal enzyme in the heme biosynthesis pathway. Mice treated with NMM accumulated PPIX instead of heme in their livers [8]. While it is difficult to determine the amount of NMM accumulated in the liver, the amount injected into mice was 80 nmols (46.4 μg or 50 μL of 1.6 mM solution). Inhibition of ferrochelatase leads to extreme light sensitivity due to the photoactivation of a singlet oxygen by PPIX (known as erythropoietic protoporphyria). Fe^2+^ insertion into planar PPIX typically proceeds *via* a transition state involving a significantly distorted porphyrin macrocycle. The crystal structure of NMM in complex with *B. subtilis* ferrochelatase confirms that NMM, with its non-planar geometry, is a stable analogue of this transition state [6].

After this discovery, NMM was used to develop nucleic acid aptamers (short sequences with high specificity for their target) *via* the *systematic evolution of ligands by exponential enrichment* (SELEX) method using the transition state analogue approach. Aptamers selected for their affinity toward NMM were expected to act as DNAzymes or ribozymes and accelerate metal insertion into MPIX, the non-methylated analogue of NMM. The majority of sequences discovered *via* SELEX were G-rich [9], and in 1996 the Sen laboratory suggested that the structural element which recognizes NMM is a GQ [10]. This conclusion was further confirmed by Sugimoto *et al.*, who reported that the 18-nt G-rich sequence 5′-GTG GGT TGG GTG GGT TGG-3′, which forms a GQ in the presence of 50 mM K^+^, binds NMM with a Ka of 1.4 μM^−1^ [11]. This sequence accelerates Zn^2+^ [11] and Cu^2+^ [12] insertion into MPIX by 2-fold and 7-fold, respectively. Yang and Browser also identified 10 aptamers, two of which bound NMM with Ka of ~1 μM^−1^ and accelerated Cu^2+^ insertion into MPIX by 2-fold [12]. Curiously, a recent report identified two NMM aptamers, Nm1 and Nm2, which were proposed to form stem-loop and GQ structures, respectively, based on biophysical evidence [13]. However, both aptamers are G-rich and GQ formation by both cannot be easily excluded. Nm1 accelerates Cu^2+^ insertion into MPIX by 3-fold and binds NMM with a Ka of 1.3 μM^−1^. Meanwhile, Nm2 accelerates Cu^2+^ insertion into MPIX by 2-fold and is a weaker NMM binder with a Ka of 0.08 μM^−1^ [13]. Thus, the ability of NMM aptamers to accelerate metal insertion (chelatase activity) seems to be positively correlated with their binding affinity for NMM. Nm2 also displayed peroxidase activity in the presence of hemin [13]. Recently, Li *et al*. developed a gold nanoparticle-based SELEX method and identified an aptamer for NMM and ZnPPIX. The aptamer showed ~7-fold binding selectivity for NMM over ZnPPIX (Ka of 0.74 *vs*. 0.10 μM^−1^, respectively) and excellent peroxidase activity. Conformational studies suggest that the aptamer forms a parallel GQ, which explains its selectivity for NMM [14].

Sen’s report influenced greatly the development of the GQ filed by identifying NMM as the first GQ-binding small molecule ligand [10].

### Optical properties of NMM alone and in complex with GQ DNA: UV-vis, fluorescence, and circular dichroism spectroscopy

**Fig. 2.**
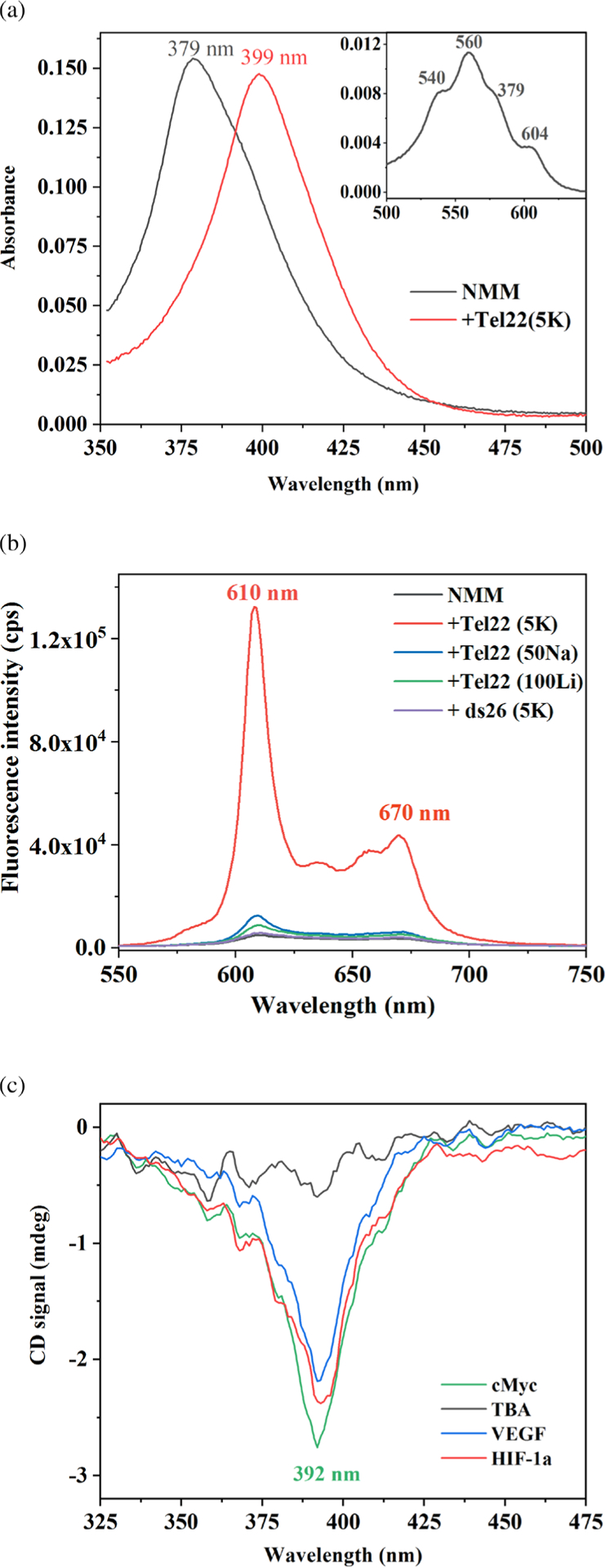
Optical properties of NMM alone and in the presence of GQ DNA in aqueous solutions. (a) Visible spectrum of 5.3 μM NMM alone and with 14.2 eq of Tel22 in the presence of 5 mM K^+^ collected in 2 mm cuvette. The major band, called the Soret band, red shifts in the presence of Tel22 from 379 to 399 nm. The inset displays Q bands of NMM. (b) Fluorescence spectra of 1 μM NMM alone and in the presence of 10 eq of Tel22 in 5 mM K^+^, 50 mM Na^+^, or 100 mM Li^+^ as well as ds26 in the presence of 5 mM K^+^. Excitation was at 399 nm, T was at 298 K, and slit width was set to 10 nm for both excitation and emission. (c) Negative iCD signal for GQ-NMM complexes at 2–4 μM in the presence of 5 mM K^+^. Note, no iCD is observed for TBA-NMM. Other sequences that lead to iCD include 26TelG4, Bcl-2, cKit1, cKit2, G4TERT, and THM G-rich sequences. Data in a and c are from [7] and in b are from [19]

The UV-vis spectrum of aqueous NMM displays a Soret band at 379 nm (ε = 1.45 × 10^5^ M^−1^ cm^−1^ [15]) and weak Q bands at 540, 560, 579, and 604 nm. Upon binding to GQ DNA, the spectrum displays a large red shift of 17–20 nm, accompanied by a relatively small change in signal intensity (hypo- or hyperchromicity) of ±20% [7] (Fig. 2a). These numbers correlate to a specific binding mode (*e.g.* intercalation or groove binding) of porphyrins to (infinitively) long dsDNA [16]. However, such correlations have not yet been established for the binding of porphyrins to GQs due to the lack of structural data.

NMM fluoresces weakly in solution when excited at 399 nm, but its fluorescence is significantly enhanced upon the addition of GQ DNA (Fig 2b). As a result, NMM can serve as a turn-on fluorescent probe [17–19]. The excitation wavelength corresponds to the Soret peak for NMM-GQ complexes (~400 nm), while NMM’s emission peaks at 610–614 nm. Our investigation into the fluorescence enhancement mechanism suggests that binding to GQs protects NMM from the solvent, thereby increasing its fluorescence lifetime [19]. We determined that NMM displays a single long lifetime (6–8 ns) in the presence of parallel GQs, but two lifetimes (5–7 and 1–2 ns) in the presence of hybrid and antiparallel GQs [19]. The fluorescence of NMM is an extremely useful property that allows for many of its applications (discussed below). The Bolton laboratory was the first to take advantage of NMM’s fluorescence to characterize both its binding to GQs and its ability to discriminate between GQ and dsDNA [18].

NMM does not display any signal in its circular dichroism (CD) spectrum because the commercially available product exists as a racemic mixture. NMM-GQ complexes, which are chiral, should produce an induced CD (iCD) signal around ~400 nm, where NMM-GQ complexes absorb. In general, the iCD signal can be positive, negative, or display a more complex pattern depending on the binding mode. While we did observe a negative iCD signal, it was weak and only appeared in the presence of some GQs (Fig. 2c) [7]. The Bolton laboratory examined 11 different NMM-GQ complexes and did not detect any iCD signal. They concluded that the absence of an iCD signal is consistent with the end-stacking binding mode (binding to terminal G-tetrads), which is associated with the lowest chirality [20]. Similarly, the Wilson laboratory observed no significant iCD signal for ethidium, a well-characterized end-stacking compound [21].

### Interaction of NMM with GQ DNA

All DNA sequences mentioned in this review are listed in Table 1.

#### Binding affinity.

NMM did not immediately capture the attention of the GQ community when its ability to bind GQ DNA was discovered, likely because of its relatively low affinity for GQs. The latter is due to the negative charge and non-planarity of NMM. Good GQ binders are usually positively charged and planar, which allows them to interact with GQs *via* electrostatic interactions and π–π stacking. One of the best porphyrin GQ binders is tetra(*N*-methyl-4-pyridyl)porphyrin (TMPyP4), which has a 4+ charge and a planar core [7]. However, TMPyP4 suffers from poor selectivity for GQ *vs*. dsDNA [15], whereas NMM does not have this fatal flaw.

When our laboratory measured the affinity of NMM toward a variant of human telomeric DNA, Tel22, in K^+^, the binding constant was low at 1 × 10^5^ M^−1^ [7]. In spite of this weak binding, we obtained crystals of NMM in complex with Tel22 and solved its structure [2]. We proceeded to investigate the binding of NMM to other GQ structures using a combination of spectroscopic methods (CD, UV-vis, Analytical Ultracentrifugation, and fluorescence titrations) and isothermal titration calorimetry (ITC). We observed a Ka of ~1.5 × 10^6^ M^−1^ for the parallel, tetrastranded T_4_G_n_T_4_ (where *n* = 4 or 8), and a much stronger Ka of ~1.3 × 10^7^ M ^−1^ for the G_4_T_4_G_4_ dimer, which is expected to form an antiparallel GQ in the presence of K^+^ [19]. Our preliminary data on NMM binding to the parallel GQs formed by VEGF, cMyc, and G4TERT yielded Ka values of >2 × 10^6^ M^−1^, suggesting strong binding (unpublished). Our most recent data on NMM binding to the parallel THM GQ in K^+^ buffer suggests a binding affinity of ~5 × 10^7^ M^−1^, the highest value determined so far for any GQ-NMM complex (unpublished). Interestingly, in Na^+^ buffers, where GQ DNA is expected to adopt an antiparallel topology, we detected no binding of NMM to G_4_T_4_G_4_, 26TelG4 [19], and Tel22 [7]. Our results (except for G_4_T_4_G_4_ in K^+^, see below) are in line with a report by Tippana *et al*., who measured the affinity of NMM for eleven GQ-forming sequences with various degrees of parallel/antiparallel topology. Their binding constants were determined by taking advantage of NMM’s ability to quench the fluorescence of Cy3 dye placed at the 3′ end of the DNA. NMM bound tightly to all parallel GQs (*e.g.* cMyc) with a Ka of ~1 × 10^7^ M^−1^, and rather weakly, with Ka lower than ~1 × 10^4^ M^−1^ to all antiparallel GQs and T_25_ single stranded DNA (ssDNA) [17].

GQ-forming aptamers developed for NMM bind with weak-to-medium affinity and Ka of (0.1–5) × 10^6^ M^−1^ [10–12]. For example, the binding affinity of NMM to the three aptamers identified by Sen and co-workers is in the range (1.3–2.5) × 10^6^ M^−1^. In this case, binding constants were determined by measuring the distribution of aptamers between free NMM in solution and NMM immobilized on a column [10]. Out of the ten aptamers identified by Yang and Bowser, two bind NMM with Ka of 0.8 × 10^6^ and 1.1 × 10^6^ M^−1^, three display Ka of (2–3) 10^5^ M^−1^, while the rest do not bind well to NMM, displaying Ka lower than 5 × 10^4^ M^−1^ [12]. The Sugimoto aptamer binds NMM with Ka of 1.4 × 10^6^ M^−1^ [12].

The binding affinities discussed are summarized in Table 2. From the table, it is apparent that NMM binds GQs with Ka spanning 2–3 orders of magnitude, ranging from (0.1–50) × 10^6^ M^−1^. While the binding of NMM to GQs is always stronger in K^+^ than in Na^+^ and could be explained by the differences in GQ topology, the cause of these large Ka differences displayed by NMM toward a variety of parallel GQs is not fully understood and requires further exploration.

#### Stabilizing ability of NMM.

We studied the stabilizing ability of NMM *via* CD melting and fluorescence resonance energy transfer (FRET) [7]. In FRET we used doubly labeled human telomeric DNA, 5′-FAM-GGG(TTAGGG)_3_-Dabcyl-3′ (F21D) and monitored FAM fluorescence as a function of temperature. The advantage of FRET is its high-throughput format which allows screening of a large number of variants (*e.g.* buffer components and ligand concentrations) in a short time and with a low amount of materials [22]. Its main drawback, however, is the possibility that ligands interact with the labels rather than the GQ itself. CD melting of unlabeled GQs in the presence of a ligand circumvents this drawback and is a direct way to determine DNA stability. While the melting temperatures derived from these two methods usually cannot be directly compared, the trends are similar.

**Fig. 3.**
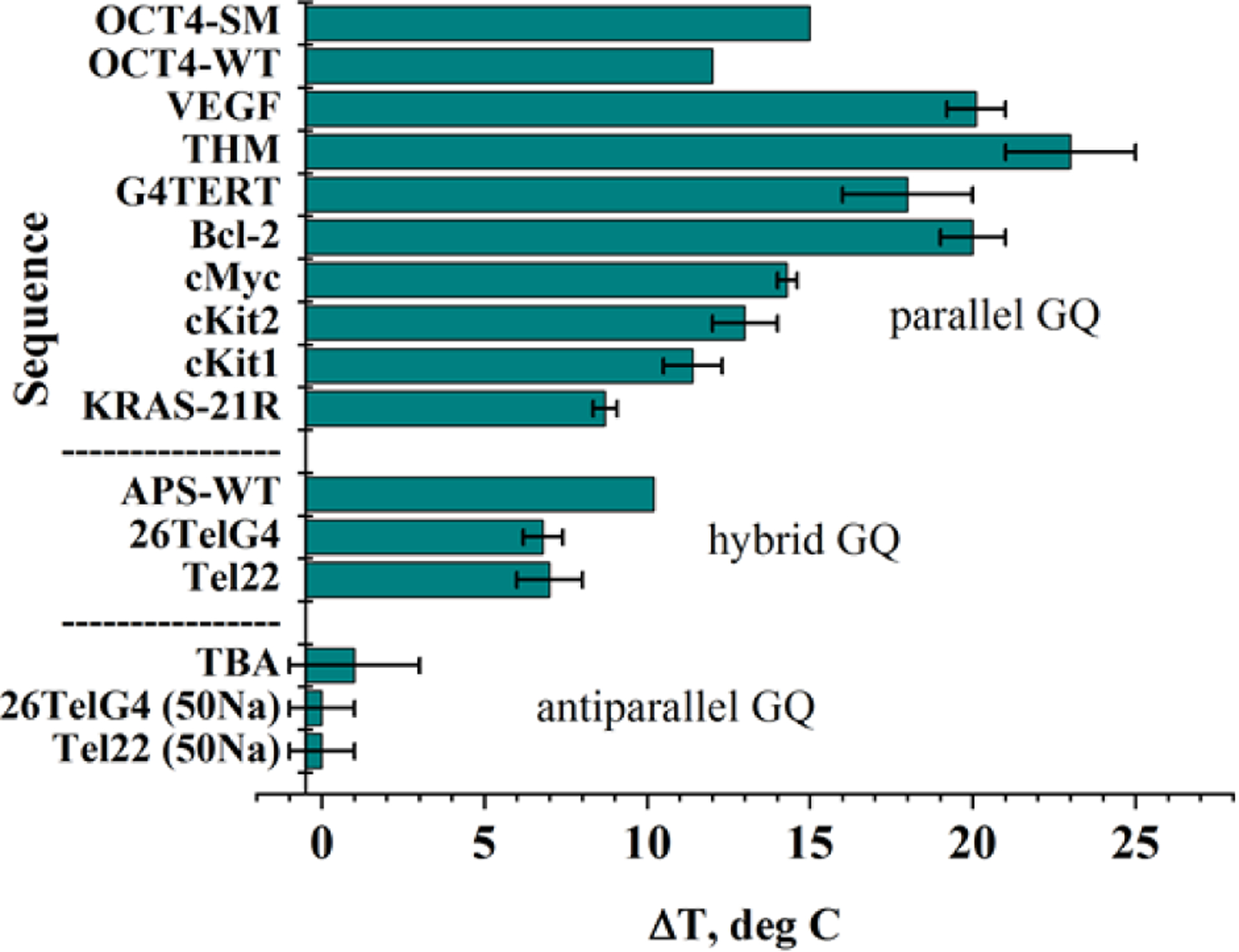
Stabilizing ability of NMM toward GQ DNA. Summary of stabilization temperatures, ΔT, determined *via* CD melting experiments in the presence of 2 eq of NMM in 5 mM K^+^. Data are from [7]. Data for KRAS-21R was collected in 20 mM KPi 6.7 and 70 mM KCl (unpublished). Data for OCT4 were collected in 10 mM KPi 7.0 and 150 mM KCl in the presence of 3 eq of NMM [70]. Data for APS-WT were collected in 1 mM NaPi 7.0, 0.3 mM EDTA, 100 mM KCl, and 2 eq of NMM [24]. Note, the specified conformation refers to GQ DNA in the absence of NMM

NMM displays excellent dose-dependent stabilizing ability toward a variety of GQ structures (Fig. 3). Consistent with results from binding studies, NMM stabilizes parallel structures and, to a lesser extent, hybrid structures, but generally does not stabilize antiparallel GQs. The presence of K^+^ is essential for stabilization, as we did not find any reports of NMM stabilization of GQs in Na^+^ buffers. Interestingly, the highest stabilization, of 23 ± 2 °C at 2 eq of NMM is observed for the THM GQ, which adopts a parallel conformation (even in the absence of NMM) and displays the highest known binding affinity for NMM (unpublished). Biologists use NMM as GQ-stabilizing treatment to determine the effect of GQs on a variety of biological processes (see “Using NMM to study GQ DNA in biological contexts”).

#### NMM-induced conformational change of G-rich DNA.

**Fig. 4.**
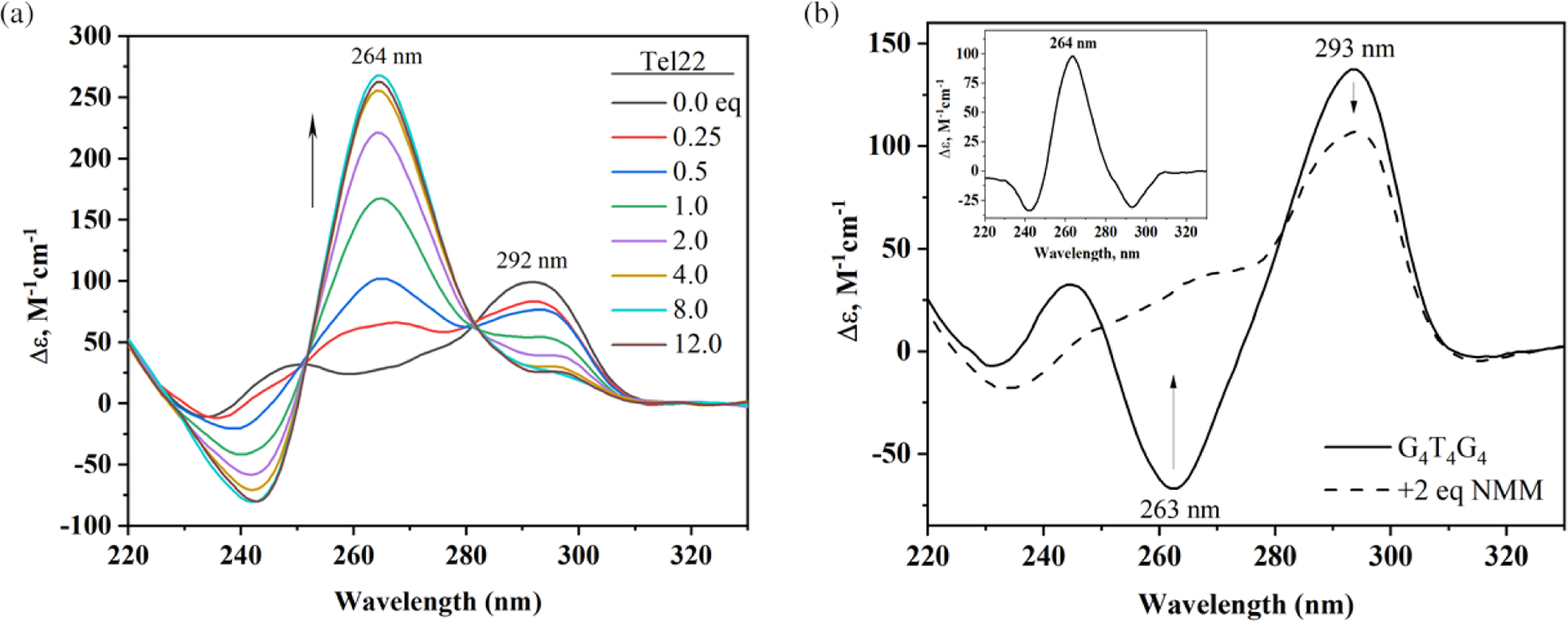
NMM-induced structural rearrangement of GQ DNA toward a parallel conformation in the presence of 5 mM K^+^ (a) Equilibrium titration of Tel22 with NMM. Figure adapted from [7]. (b) CD signal for G_4_T_4_G_4_ alone and in the presence of 2 eq of NMM. The inset shows the CD signal difference induced by NMM. Figure adapted from [19]. Parallel and antiparallel GQ conformations are signified by the CD signal at 264 and 292 nm, respectively. All data were collected under thermodynamic control, *i.e.* DNA in each CD scan was annealed with the indicated amount of NMM, slowly cooled, and incubated for >12 h at 30 °C for (a) and 4 °C for (b)

In the process of characterizing NMM binding to Tel22, we discovered that NMM is capable of inducing a structural transition from hybrid to parallel conformation, as signified by the increase in CD signal at 264 nm (Fig. 4a). This conversion is slow, occurring over ~30 h at 30 °C in 5 mM K^+^ [7]. We hypothesize that the NMM-induced structural rearrangement of Tel22 happens *via* conformational selection mechanism described by the Chaires group for the Tel22-PEG (polyethylene glycol) system [23]. In essence, Tel22 in K^+^ exists as an equilibrium mixture of multiple conformations, one of which is parallel, estimated at ~14%. NMM preferentially binds to the parallel conformation, which shifts the equilibrium, until full conversion occurs. We observed similar structural transitions for Bcl-2, G4TERT, cKit1, (TTGGGG)_4_, and G_4_T_4_G_4_ (Fig. 4b), among others [7, 18, and unpublished]. Such conversions are not observed in Li^+^ or Na^+^. For DNA that adopts a parallel GQ geometry by itself, NMM either increases the signal intensity at 264 nm or does not change the CD signal. A recent study by the Renčiuk lab [24] demonstrated that 2 eq of NMM induces parallel conformation in two human telomeric sequences, (GGGTTA)_3_GGGT and Tel22, and one model sequence, APS-WT, all of which adopt antiparallel/hybrid conformations in K^+^ buffer. The effects of NMM on GQ DNA are similar to, but somewhat weaker, than those of 60% PEG200 or 60% ethanol. The ability of NMM to modulate GQ structures must be taken into consideration in biological studies.

#### Selectivity of NMM for GQ DNA vs. other DNA structures.

A variety of experimental approaches showcase NMM’s selectivity, including competition dialysis [25, 26], fluorescence enhancement [19, 20, 27], FRET melting [7], single-molecule FRET (smFRET) [17], and helicase inhibition assays [28, 29]. All these studies conclude that NMM is exceptionally selective for GQ DNA over other types of DNA, including ssDNA, dsDNA, DNA-RNA hybrids, dsRNA, Z-DNA, and triplex DNA [15, 18, 19, 25].

**Fig. 5.**
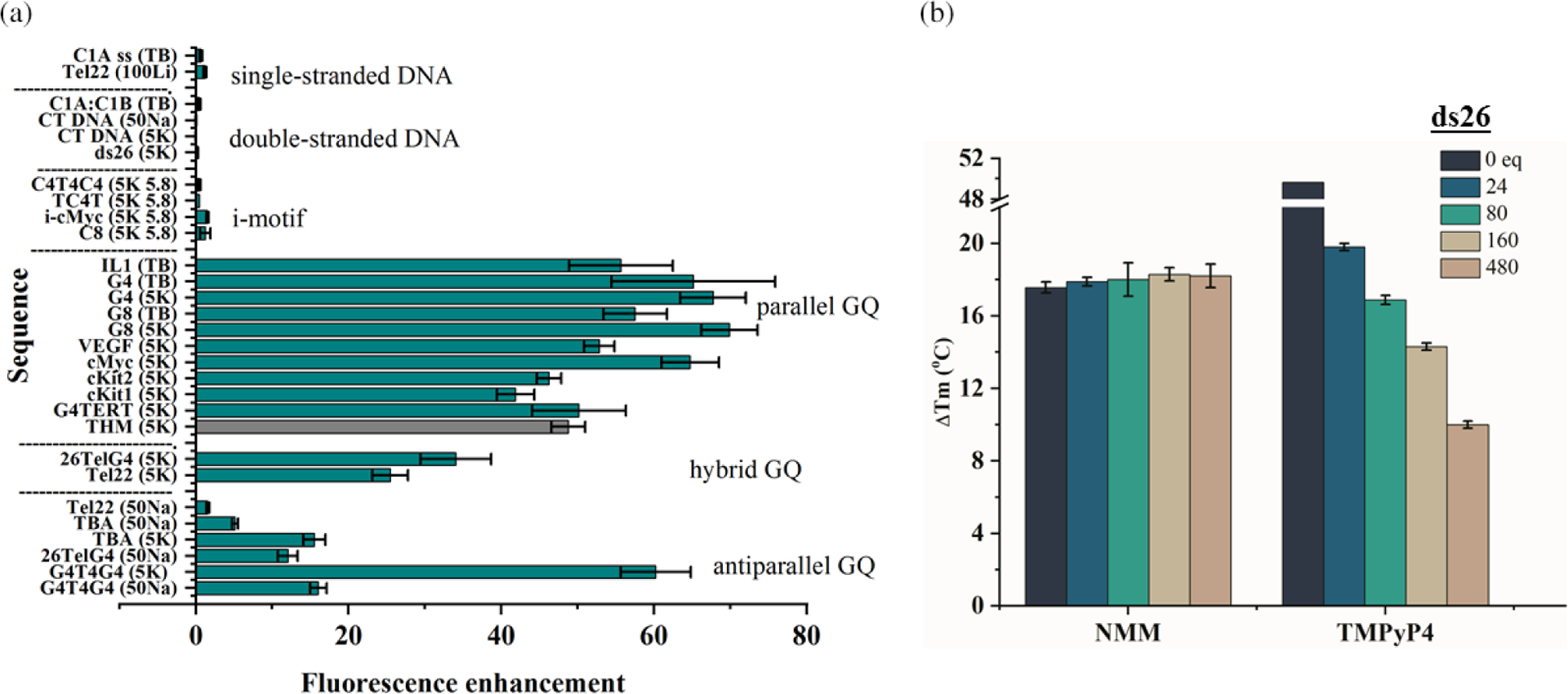
Selectivity of NMM for GQ DNA. (a) Fluorescence enhancement data for NMM in the presence of 10-fold excess of the indicated DNA structures. Data for the THM sequence (in grey) was collected in the presence of 2–5 eq of DNA (unpublished). Buffers are the same as in Table 2. In addition, 100Li is 10 mM lithium cacodylate 7.2, 100 mM LiCl; 5K 5.8 is 5K buffer adjusted to pH 5.8. Figure adapted from [19]. (b) Stabilization of 0.2 μM F21D, 5′-FAM-GGG(TTAGGG)_3_-Dabcyl-3′, by 1.6 μM NMM and TMPyP4 in the presence of the specified amount of dsDNA competitor, ds26, in 5 mM K^+^ determined in FRET assay. Figure adapted from [7]

In competition dialysis, fluorescent enhancement, and smFRET, selectivity was assessed by looking at NMM’s response in the presence of different DNA topologies (Fig. 5). When the response was high, strong NMM-DNA interaction was assumed. By quantifying equilibrium dialysis data, Chaires concluded that NMM binds to GQs more favorably by at least 2 kcal/mol as compared to other tested DNA structures [25]. In FRET melting studies the ability of NMM to stabilize doubly labeled human telomeric GQ DNA, F21D, was tested in the presence of a large quantity of dsDNA competitors. The stabilizing ability of NMM, unlike that of TMPyP4, was not compromised even when in the presence of a large excess of dsDNA (Fig. 5b), which is indicative of its excellent selectivity. NMM’s selectivity was also demonstrated in helicase inhibition assays using the RecQ family helicases, BLM (human), Sgs1p (*Saccharomyces cerevisiae*) [28], and RecQ (*Escherichia coli*) [29]. DNA helicases are ATPases that remodel DNA and DNA-protein complexes. NMM selectively inhibits unwinding of GQs by BLM and Sgs1p with an inhibition constant, K_i_, of ~0.8 and ~1.0 μM, respectively, but not dsDNA or Holliday Junctions (K_i_ of ~25 μM) [28]. The observed inhibition was likely caused by NMM’s ability to stabilize GQs but not other DNA structures. The data also demonstrate that NMM affects neither helicase binding to DNA nor the ATP hydrolysis steps. On the other hand, the porphyrin ligand TMPyP4 inhibits BLM and Sgs1 activity toward both GQ and dsDNA without discrimination [28], in line with the poor selectivity of this ligand.

Finally, we would like to mention NMM’s selectivity toward i-motif DNA, an intercalated, hemi-protonated cytosine structure [30]. The literature reports are scarce and without agreement. The Chaires laboratory reported binding of NMM to bimolecular C_4_T_4_C_4_, tetramolecular TC_4_T, and poly(dC) i-motifs *via* the equilibrium dialysis method [25]. We investigated the first two sequences, as well as i-cMyc and A_4_C_8_A_4_, using fluorescence enhancement of NMM, and observed no binding (Fig. 5a) [19]. Further investigation is required to resolve this discrepancy.

#### Selectivity of NMM for parallel GQs vs. other GQ topologies.

NMM displays excellent selectivity for parallel GQs — a highly desirable but rare property among GQ ligands, which often interact with a broad range of GQ conformations. Using ~10 G-rich DNA sequences each, the Chaires and Bolton laboratories discovered that NMM displays a clear preference for some GQ structures over others [20, 25]. However, they made no attempts to correlate this preference with the exact GQ topology. Later, Tippana *et al*. reported a strong correlation between NMM’s binding affinity and the percentage of parallel GQ character measured *via* smFRET, which clearly indicated NMM’s preference for the parallel GQ topology [17]. Our laboratory arrived at a similar conclusion while working with Tel22. NMM’s fluorescence increases only slightly in the presence of unfolded Tel22 in Li^+^ or antiparallel Tel22 in Na^+^, but increases by 25-fold in the presence of hybrid Tel22 in K^+^ (Fig. 2b) [7, 19]. We then expanded this study to include ~20 different GQs (Fig. 5a) and determined that NMM fluorescence increases by ~60-fold in the presence of parallel GQ structures, ~40-fold in the presence of hybrid structures, and ~10-fold in the presence of antiparallel structures (excluding the unusual case of G_4_T_4_G_4_) [19]. The latter DNA is expected to form a bimolecular antiparallel GQ in both K^+^ and Na^+^ [31, 32], so the fluorescence enhancement was expected to be low. Contrary to our expectations, however, the addition of G_4_T_4_G_4_ to NMM in the presence of K^+^ led to fluorescence enhancement of 60 ± 5%, equal to that of many parallel sequences (Fig. 5a). We explain this result, at least in part, by NMM-induced structural conversion of G_4_T_4_G_4_ into a conformation with increased parallel nature, as judged by the increase in the CD signal at 264 nm, which is characteristic for parallel GQs (Fig. 4b). The Chaires laboratory also observed a strong preference of NMM for a related sequence (G_4_T_4_)_3_ [25]. A crystal or NMR structure of NMM in complex with the G_4_T_4_G_4_ GQ may help explain the strong preference of NMM toward this (presumably) antiparallel structure.

In summary, NMM demonstrates unparalleled selectivity for GQs over other DNA secondary structures, albeit with a lower affinity than other small molecule ligands. Among GQs, it strongly prefers the parallel conformation, followed by the hybrid, and only weakly interacts (if at all) with antiparallel GQs.

#### The end-stacking binding mode of NMM to GQ DNA.

**Fig. 6.**
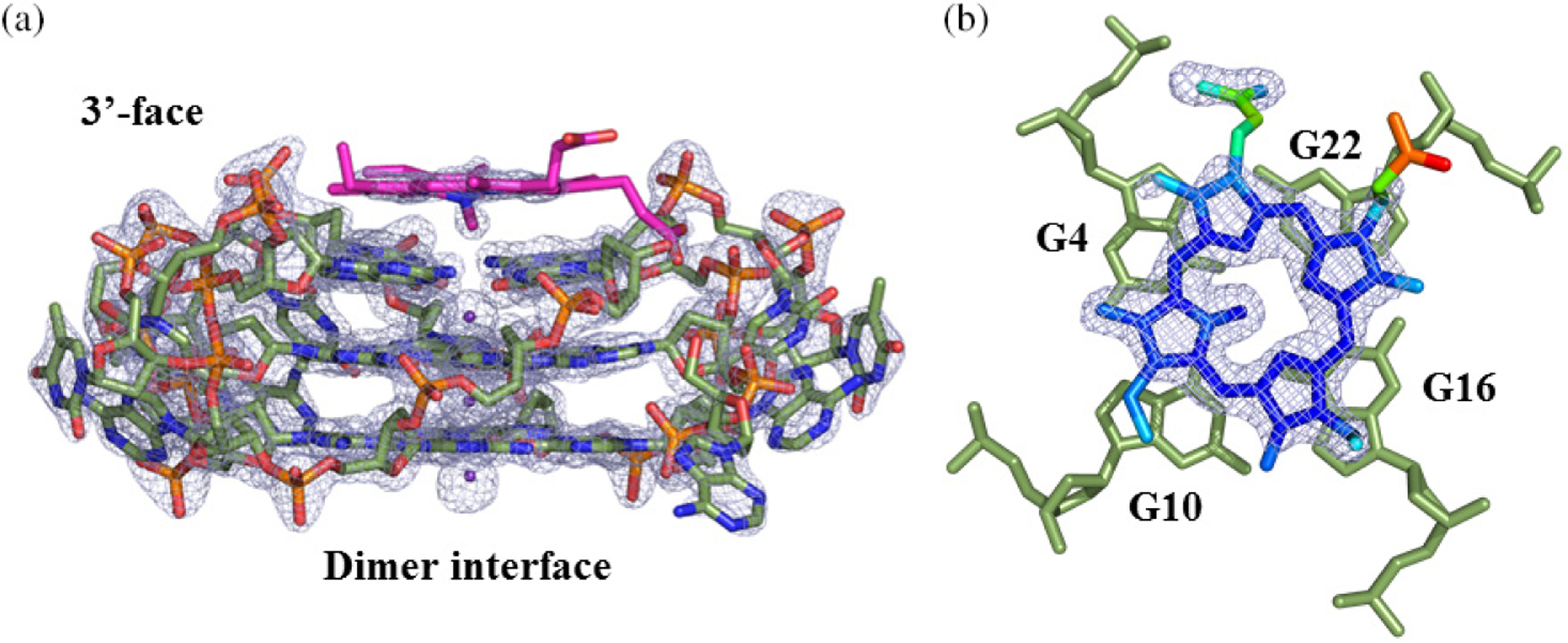
Tel22-NMM complex at 1.65 Å (PDB ID 4FXM). (a) Model of the Tel22-NMM complex at 1.65 Å (PDB ID: 4FXM) embedded in an electron density map drawn at 2σ. NMM is shown in magenta. (b) Structure of NMM color coded for its temperature factors (lowest — blue; highest — red) and corresponding electron density map at 1σ. The blue color of the core suggests that it is well defined, while red color of one propionate group indicates that its position is not certain. Adapted with permission from (Nicoludis JM, Miller ST, Jeffrey PD, Barrett SP, Rablen PR, Lawton TJ and Yatsunyk LA. *J. Am. Chem. Soc.* 2012; **134**: 20446–20456). Copyright (2019) American Chemical Society

Intercalation of NMM into GQs is a myth. Neither spectroscopic nor structural studies point to the ability of NMM to intercalate between G-tetrads by overcoming their efficient π–π stacking interactions. In fact, no GQ ligand to date has been shown to intercalate into a short biological GQ (with 2–4 G-tetrads) *via* structural methods. Instead, our crystal structure of the NMM-Tel22 complex, solved to 1.65 Å (PDB ID: 4FXM), indicates that NMM stacks onto the 3′ G-tetrad (*i.e.* end-stacking binding mode) (Fig. 6) [2]. The NMM macrocycle is positioned 3.6 Å away from the 3′ G-tetrad, which is consistent with the π–π stacking distance observed for a variety of GQ-ligand complexes (ligands: berberine, metal-salphene, and naphthalene diimide) [33–35]. The end-stacking binding mode is a common way for (nearly) planar ligands to interact with GQ DNA. In the NMM-Tel22 crystal structure, the *N*-methyl group of NMM is bent away from the mean macrocycle plane by 44.8° and points into the potassium channel in the center of the Tel22 GQ (Fig. 6a). This arrangement leads to the off-centered placement of NMM toward one of the guanines in the 3′ G-tetrad, G_22_ (Fig. 6b). While the geometry of the GQ is invariant in its free and ligand bound forms, the efficiency of binding can be explained by the ability of NMM to adjust its geometry to match that of the terminal G-tetrad to which it binds. Furthermore, efficient π–π stacking and complementarity between the surface of 3′ terminal G-tetrad and NMM can explain the excellent selectivity of NMM for parallel GQs. Meanwhile, the smaller Na^+^ ion usually occupies the space within a terminal G-tetrad [36], preventing the N-Me group from entering the ion channel and thereby inhibiting binding. The electron density in our structure is not clear enough to support with confidence Tel22’s preference for one NMM isomer. On the contrary, only one NMM isomer, with the methyl group on pyrrole ring A (Fig. 1a), was shown to bind to *B. subtilis* ferrochelatase [5], suggesting stereoselectivity. The His183Ala mutant of *B. subtilis* ferrochelatase had preference for another NMM isomer, with the methyl group on ring B [6]. Yet all four isolated NMM isomers equally inhibited the enzyme [37]. Recently, our laboratory solved the crystal structure of NMM in complex with THM at 2.4 Å (PDB ID: 6PNK and 6P45). The structure shows great resemblance to that of the NMM-Tel22 complex.

**Fig. 7.**
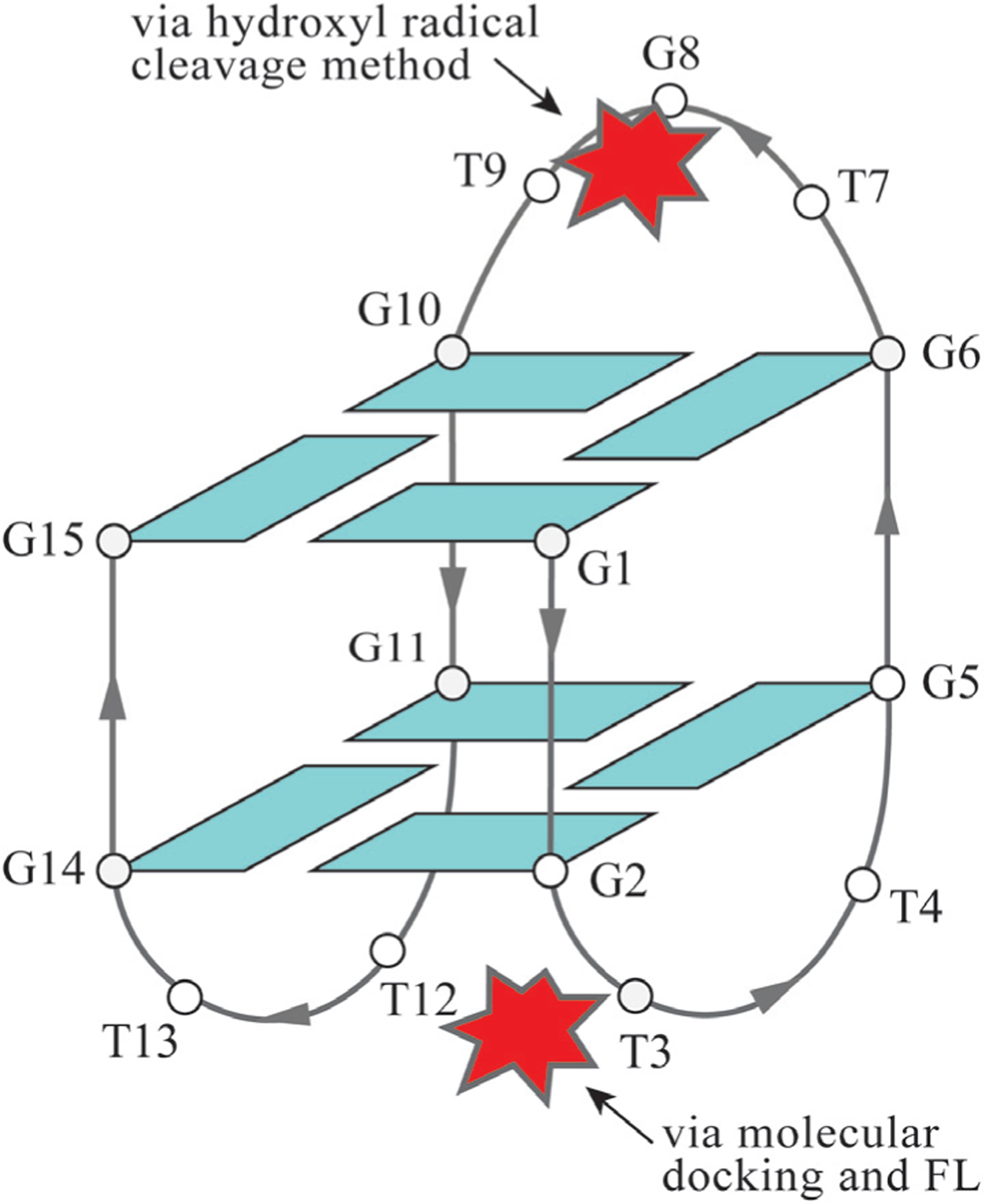
Interaction of the Thrombin binding aptamer, TBA, with NMM determined by biophysical and computational methods. TBA forms an antiparallel structure in both K^+^ and Na^+^ conditions. Two different possible binding modes of NMM, depicted as a red star, are shown. Hydroxyl radical cleavage was run in a buffer which contained 100 mM NaCl and 10 mM KCl

While no other structures (NMR or X-ray) of NMM-GQ complexes exist, the binding mode of NMM can also be inferred from spectroscopic and footprinting experiments, as well as from computational studies. Two reports attempted to determine the binding mode of NMM to the thrombin-binding aptamer, TBA, a widely studied G-rich sequence which forms an antiparallel GQ (Fig. 7). The TBA GQ has anticoagulant properties due to its nanomolar affinity for exosite I of human α-thrombin. In the first report, the enhanced hydroxyl radical cleavage method was used to demonstrate that NMM binds to TBA by forming a bond with a single base in the central (top) TGT loop [38]. The hydroxyl radical cleavage protocol can detect any base with an exposed sugar. Ligand binding sites are determined by comparing the cleavage patterns for DNA and ligand-DNA complexes. The second report used NMM and Thioflavin T (ThT) fluorescence, coupled with molecular docking experiments, to conclude that NMM interacts with TBA at the opposite end of the GQ, which contains two TT loops [39]. The propionic acid side chains of NMM are proposed to form hydrogen bonds with one guanine from the bottom G-tetrad and one thymine from the TT loop. Two additional thymines and one guanine π–π stack onto NMM, but this binding is weaker. Interestingly, the authors did not observe binding of NMM to an unusual parallel form of TBA formed in the presence of ThT (and absence of K^+^). The authors explained this lack of binding as being due to the two TT loops being far apart in this conformation. We can propose an alternate explanation: NMM’s lack of interaction with TBA could also be due to its binding site being already occupied by ThT. Alternatively, in our experience, NMM always binds weakly or not at all to GQs formed in the absence of K^+^. While contradictory in terms of the specific binding interactions, the two reports indicate that NMM does not end-stack onto the terminal G-tetrad in TBA. This explains why our laboratory observed low fluorescence enhancement of 16 ± 1% in K^+^ and 5.2 ± 0.4 in Na^+^ (Fig. 5a) [19], as well as no stabilization of TBA by NMM (Fig. 3) [7].

In summary, structural studies indicate that NMM binds to Tel22 [2] (PDB ID: 4FXM) and THM (PDB ID: 6PNK and 6P45) GQs *via* end-stacking to the 3′ G-tetrad. Biophysical analysis of NMM binding to TBA reports that NMM interacts with either the top loop [38] or the bottom loops [39] (Fig. 7). Further studies are needed to resolve this discrepancy.

### NMM-GQ complexes as fluorescent sensors

**Fig. 8.**
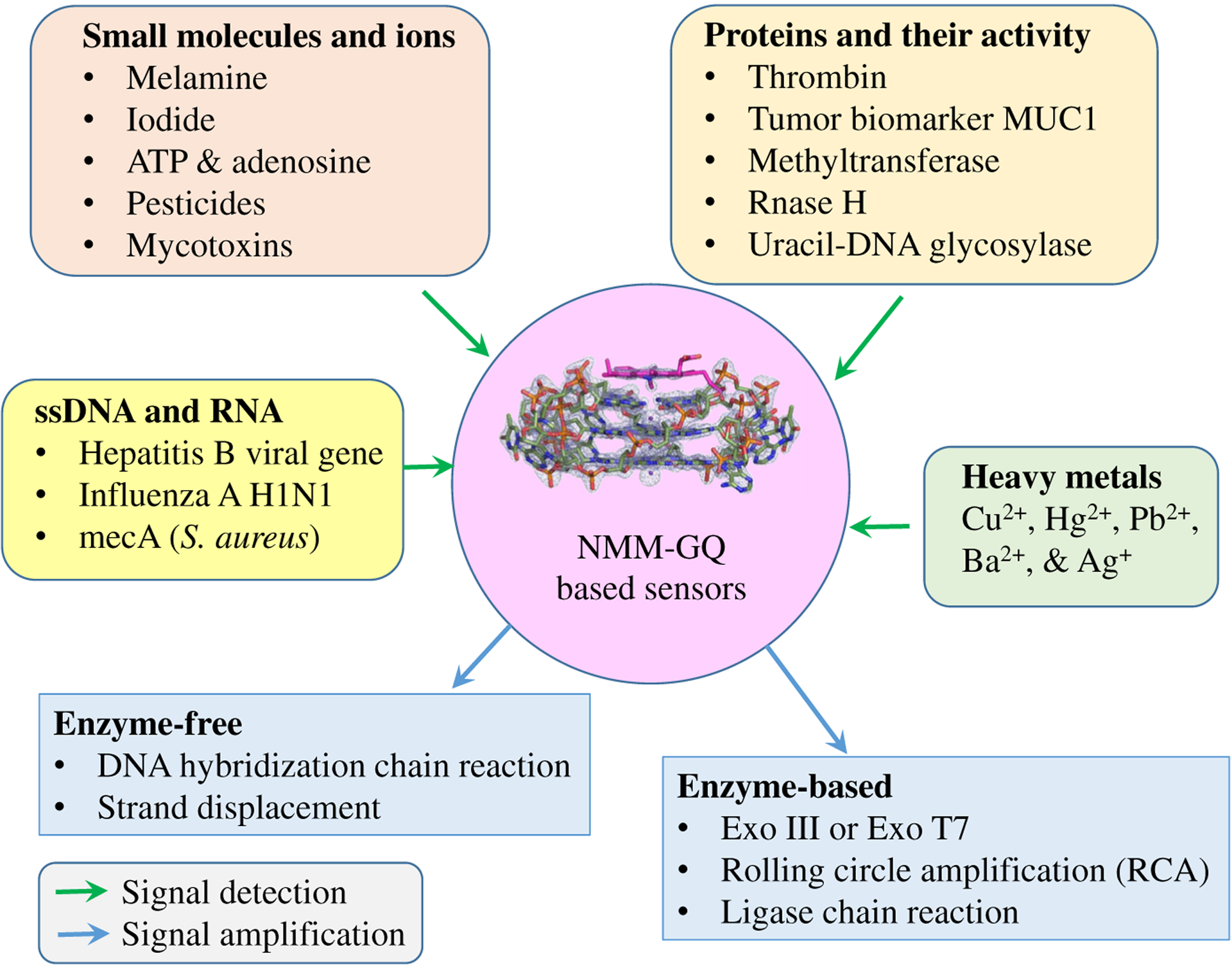
Summary of the variety of NMM-GQ based sensors and the molecules they are designed to detect. The detection step for turn-on sensors involves quantitative increase in NMM fluorescence in response to target-induced GQ formation. The detection step for turn-off sensors involves quantitative decrease in NMM fluorescence due to target-induced unfolding of the GQ. Amplification steps can be subdivided into enzyme-based and enzyme-free. Both types lead to a dramatic increase in sensitivity of the sensors

The exceptional selectivity of NMM for GQ DNA allows for its wide applications in chemistry and biology. Specifically, NMM-GQ complexes can serve as label-free turn-on or turn-off sensors whose fluorescence is proportional to the concentration of an analyte. In all turn-on sensors, the addition of an analyte triggers GQ formation or stabilizes an existing GQ, which then interacts with NMM, thereby leading to an increase in fluorescence. Meanwhile, in turn-off sensors, highly fluorescent NMM-GQ systems are disrupted by the analyte due to remodeling or unfolding of the GQ, or alternatively due to specific interactions with the side chains of NMM. NMM-GQ sensors provide a simple, cost effective and highly sensitive method for identifying a range of analytes that includes heavy metals, anions, small molecules (including ATP and pesticides), DNA, and proteins. Their dynamic range can be controlled by the precise selection of G-rich DNA fragments (which lead to varied fluorescence responses, see Fig. 5a) and buffer components (notably K^+^). Sensor sensitivity can be further improved by incorporating enzyme-based signal amplification strategies (such as rolling circle amplification (RCA), ligase chain reaction, and exonuclease amplification) or enzyme-free technologies based on hybridization chain reaction and strand displacement. The most common amplification strategy relies on the ability of exonuclease to digest blunt 3′ (Exo III) or 5′ (Exo T7) ends of dsDNA making this method extremely general and allowing detection and signal amplification for any analyte as long as its DNA binding partners can be identified (*e.g.* aptamers or complementary sequences). NMM-GQ sensors described in literature are summarized in Fig. 8 and discussed below.

#### Detecting heavy metal ions.

Heavy metals have great utility in various industries, but their toxicity can adversely impact both the environment and human health. Although it is important to be able to detect heavy metals, established methods are expensive, time-consuming and complicated. Accordingly, new and improved methods are highly sought after.

Two studies exploited the fluorescence enhancement upon NMM’s interaction with a G-rich sequence called AGRO100 to detect Ba^2+^ [40], Pb^2+^, and Hg^2+^ [41]. In the first study, a turn-on probe was developed based on the ability of Ba^2+^ to induce AGRO100 folding into a GQ, which is detected by NMM with a limit of 4 nM [40]. This value is 3600 times lower than the maximum allowed Ba^2+^ concentration in drinking water as defined by the U.S. Environmental Protection Agency (EPA). Other metals that promote AGRO100 folding into a GQ (*e.g.* K^+^, Pb^2+^) may interfere with this assay. This system was then converted into a turn-off detector for Pb^2+^ and Hg^2+^ [41]. NMM displays high fluorescence when AGRO100 is folded into a GQ in a K^+^ buffer. Addition of Hg^2+^ leads in T–Hg^2+^–T interactions, which change the GQ into a hairpin structure that NMM does not bind to. Similarly, addition of Pb^2+^ converts the GQ into a new conformation that no longer interacts with NMM. For Pb^2+^ detection, Hg^2+^ interference was eliminated by adding polythymine (T_20_), which sequesters Hg^2+^ by forming a hairpin. 2,6-pyridinedicarboxylic acid was added to eliminate interference from Pb^2+^ when detecting Hg^2+^. The sensitivity for Pb^2+^ is 5 nM and the sensitivity for Hg^2+^ is 18.6 nM [41]. A similar turn-off Pb^2+^ sensor based on another G-rich sequence called PS2.M was reported by Guo *et al*. with a 1 nM detection limit [42].

A turn-off sensor for Cu^2+^ (detection limit of 80 nM) was developed by Qin *et al*. using the 24GT GQ-forming sequence [43]. In the absence of Cu^2+^, the NMM-24GT complex is highly fluorescent. However, Cu^2+^ quenches fluorescence of NMM by binding to its carboxylate groups. Cr^3+^ and Fe^3+^ interfere with the assay, but addition of ssDNA sequesters these metals and eliminates interference.

A turn-on Ag^+^ sensor relies on the ability of Ag^+^ to interact with cytosine (C) bases, forming C–Ag^+^–C, and uses the Exo III signal amplification strategy [44]. Addition of Ag^+^ to a hairpin probe creates a 3′ blunt end *via* the formation of C–Ag^+^–C. Exo III then digests this blunt end, releasing Ag^+^ (to start a new cycle), along with a trigger DNA sequence. This trigger binds to a G-rich signal sequence to create a new blunt end, which Exo III digests. Upon digestion, the trigger DNA is released to start a new cycle, while the G-rich sequence folds into a GQ and binds NMM, leading to fluorescence enhancement. This Ag^+^ biosensor, with its detection limit of 2 pM, is comparable to conventional methods of Ag^+^ detection (such as atomic absorption spectroscopy, ICP-MS, and the ion-selective electrode), but does not require any specialized instrumentation.

In summary, Cu^2+^, Hg^2+^, Pb^2+^, Ba^2+^, and Ag^+^ biosensors were developed based on the fluorescence of NMM-GQ complexes. These sensors have excellent selectivity and sensitivity, with detection limits well below those defined by the World Health Organization (WHO) or EPA for drinking water. Their dynamic range can be tuned to span the concentrations desired by either adjusting the amount of NMM or using G-rich sequences with varying affinities for NMM.

#### Detecting melamine and iodide.

Melamine (Mel), an industrial chemical, is often used in dinnerware, adhesives, coatings, and flame retardants. It is used illegally to enhance the apparent protein levels in foods. A Chinese milk scandal brought world attention to the toxic effect of this chemical in 2008. Two turn-off Mel sensors were developed utilizing the ability of Mel to form a hydrogen bonded T–M–T triad [45, 46]. In this sensor, the G-rich part of the DNA is flanked by TTTTT stretches at the 5′ and 3′ ends. In the absence of Mel, the sensor adopts a GQ conformation and binds NMM. Addition of melamine unfolds the GQ to form a hairpin with a T–Mel–T stem. The excellent sensitivity of 80 nM [45] is further improved to 25 fM using the Exo III signal amplification and strand displacement strategies [46]. The sensor has proven successful when applied to real milk samples.

The turn-off Mel sensor was converted to a turn-on iodide sensor [45]. The starting state of this sensor is a hairpin with five T–Hg^2+^–T base pairs in the stem. Addition of I^−^ removes Hg^2+^ (in the form of HgI_2_ and [HgI_4_]^2−^) freeing the G-rich part to form a GQ that binds NMM. The detection limit of this biosensor is 4.6 nM, which is below the acceptable I^−^ levels defined by the WHO.

#### Detecting ATP and adenosine.

The ATP sensor consists of NMM, a DNA strand composed of an ATP aptamer followed by a sequence complementary to a blocking probe, the blocking probe, and two DNA hairpins (H1 and H2), one of which can fold into a GQ [47]. This system uses an enzyme-free signal amplification strategy based on a hybridization chain reaction involving H1 and H2. Each ATP molecule releases one blocking probe, which initiates a hybridization chain reaction that leads to the formation of long, GQ-decorated, DNA nanowires. NMM binds to these GQs, resulting in high fluorescence. In principle, any analyte can be detected by incorporating an appropriate aptamer while keeping the rest of this sensor unchanged. The specificity of the sensor is mostly defined by the specificity of the aptamer. Analysis of ATP in urine was successfully conducted using this NMM-GQ-based biosensor [47].

The adenosine sensor was developed based on an adenosine aptamer [48]. In the presence of adenosine, dsDNA containing the adenosine aptamer as one of its strands dissociates, releasing ssDNA that is complementary to the stem of a DNA hairpin with a G-rich loop. Hybridization of the ssDNA with the DNA hairpin opens the hairpin, thereby enabling GQ formation followed by NMM binding and fluorescence enhancement. Both steps are amplified using the T7 Exo-based dual recycling signal amplification strategy leading to a detection limit of of ~1 μM.

#### Detecting pesticides and mycotoxins.

A highly sensitive turn-off NMM-GQ sensor was developed by the Wang lab to detect carbamate and organophosphorus pesticides in agricultural products [49]. These pesticides inhibit acetylcholinesterase (AChE) activity in the nervous system and can thus be fatal. The sensor is designed such that in the absence of a pesticide, a normal activity of AChE will lead to the release of a trigger DNA, which initiates a hybridization chain reaction resulting in the formation of a long, GQ-decorated duplex. These GQs are recognized by NMM, leading to a fluorescence enhancement. The presence of a pesticide inhibits AChE, which decreases the amount of trigger DNA, and, accordingly, NMM fluorescence decreases.

Recently, a NMM-GQ biosensor was developed to detect an ochratoxin A [50], one of the most abundant mycotoxins implicated in nephrotoxicity and renal tumors.

#### Detecting ssDNA and RNA.

Disease diagnosis and genetic screening require quick and reliable detection of ssDNA. NMM-GQ based sensors for ssDNA all have similar designs but utilize different GQ-forming sequences or amplification strategies [51–55]. These sensors are modular and can be easily redesigned to detect *any* desired target by replacing the DNA sequence complementary to the target. In general, the presence of one base mismatch leads to >30% signal reduction. The sensors discriminate completely against DNA targets with 2–3 mismatches and can function in the high background of non-complementary DNA. We highlight specific examples of DNA biosensors below.

A sensor developed by the Hu laboratory involves a single DNA strand that consists of three parts in the following order: a GQ-forming part, a part complementary to a target DNA, and a blocking part that is complementary to a portion of the GQ-forming part. In the absence of the target, the biosensor exists as a hairpin whose loop is complementary to the DNA target. When the DNA target is added, it hybridizes with the hairpin loop, pulling the two ends of the probe apart and releasing the G-rich part to fold into a GQ in the presence of K^+^, thereby leading to enhanced NMM fluorescence [51].

Two ssDNA sensors were developed based on split G-rich DNA sequences. The idea behind these sensors is that splitting a G-rich DNA sequence into two separated parts will prevent GQ formation, leading to low NMM fluorescence. Only upon addition of the target DNA do the two parts come together, restoring GQ and resulting in high NMM fluorescence. The first sensor consists of two DNA strands, each of which has half of the cMyc G-rich DNA along with a region complementary to the target DNA, the hepatitis B viral (HBV) gene [52]. Both K^+^ and HBV are required to restore the GQ structure, which leads to high NMM fluorescence. The second sensor consists of an oligonucleotide (P1) with the sequence dG_3_T_4_G_3_-loop-G_3_T_4_G_3_ (loop = 16-nt), as well as a 22-nt oligo (P4) that is fully complementary to the target and 16-nt loop of P1. The target DNA removes P4 *via* strand displacement (as P4 and the target share more complementary bases than P4 and P1), thereby freeing P1 to fold into a GQ that binds NMM [54]. The detection limit is 2.3 nM, which is comparable to other DNA detection methods.

Li *et al*. designed an influenza A H1N1 turn-off sensor that is composed of (i) a GQ-forming part extended by a sequence complementary to a portion of the H1N1 DNA target (QBF-DNA); and (ii) an assistance DNA complementary to parts of both the QBF-DNA and H1N1 DNA [53]. In the absence of H1N1, the QBF-DNA forms a GQ which causes high fluorescence of NMM. When present, the H1N1 target hybridizes with both the QBF-DNA and assistance DNA, unfolding the GQ and forming a three-way junction, turning off the fluorescence of NMM. This biosensor has an impressive 8 pM detection limit, and its feasibility was demonstrated by the detection of H1N1 DNA in patient sera.

An interesting strategy was developed that allows for detection of *any* ssDNA. It consists of Exo III, NMM, and a DNA duplex with a G-rich segment and 3′ overhang that makes it stable in the presence of Exo III [56]. The addition of a target DNA creates a 3′ blunt end and initiates Exo III digestion of the original duplex. This process releases both the G-rich segment and the target DNA itself. The former folds into a GQ and enhances NMM fluorescence, while the latter is free to hybridize with another biosensor to amplify the signal. The reported sensitivity is 35 pM. The described strategy was used in the design of a biosensor for the mecA gene in *Staphylococcus aureus*, a foodborne bacteria with severe adverse effects on animal health. It has an impressive sensitivity of 2.4 fM [55].

NMM-GQ based biosensors can also be engineered to detect RNA. For example, Yan *et al*. designed a sensor for microRNA (miRNA), a short, non-coding, endogenously expressed RNA involved in negative post-transcriptional regulation. The sensor uses strand displacement as a signal amplification strategy and consists of NMM, a duplex-specific nuclease (DSN), and cDNA/G-rich DNA duplex [57]. The cDNA is complementary to the target miRNA and partially complementary to the G-rich DNA. When the miRNA target is present, it hybridizes with the cDNA *via* strand displacement, releasing the G-rich DNA to form a GQ that interacts with NMM, enhancing its fluorescence. The signal is amplified in the presence of the DSN, which digests the cDNA strand of the cDNA/miRNA heteroduplex, releasing the miRNA to hybridize with additional biosensors and continue the cycle. Impressively, this sensor can differentiate one miRNA (mi-141) from four highly similar members of the same miR-200 family, which was recently identified as one of the most promising targets for anticancer therapy [58].

In summary, a variety of ssDNA biosensors take advantage of NMM-GQ fluorescence as a detection method. These sensors display sensitivity equal to or better than that of other ssDNA detection techniques and provide scientists with a rich toolbox for detecting a variety of ssDNA targets. The appropriate choice of G-rich sequences along with amplification strategies can push the detection limits to even lower values.

#### Detecting proteins.

NMM, in combination with ThT, was reported to detect thrombin, an essential enzyme in hemostasis [39]. Both ligands have similar excitation wavelengths (399 and 420 nm, respectively), so they can be excited simultaneously, but have different emissions (610 and 487 nm, respectively), so they can be detected separately. The biosensor consists of NMM, ThT, and TBA and displays strong ThT fluorescence due to its interaction with TBA. Addition of thrombin induces the formation of antiparallel TBA GQ, leading to increase in NMM fluorescence. This observation is in line with our measurements of fluorescent enhancement of 16 ± 1% (Fig. 5a) for NMM-TBA in K^+^ buffer [19]. The fluorescence intensities of NMM and ThT have a linear relationship when the thrombin concentration is between 0 and 40 μM. This assay can accurately measure thrombin levels above 0.24 μM [39], rendering it potentially useful for monitoring changes in thrombin levels at the upper end of the range for blood clotting (full range: 0.1 nM–0.5 μM [59]).

A general strategy for detecting protein biomarkers relies on aptamer recognition of the target and Exo III signal amplification. First, magnetic beads decorated with the aptamer are hybridized with a ssDNA. Binding of the target protein releases the ssDNA, which hybridizes with a G-rich hairpin to create a 3′ blunt end. Exo III then digests the blunt end, releasing both the G-rich part of the hairpin (which folds into a GQ and enhances NMM fluorescence) and the ssDNA (allowing it to open another hairpin and amplify the signal). The biosensor is highly versatile and can be readily engineered by incorporating appropriate aptamers to detect many disease-related proteins. For example, MUC1, a tumor biomarker highly expressed in human adenocarcinomas, was detected with a detection limit of 3.7 nM [60].

Another GQ-NMM-based sensor was developed to detect DNA methyltransferase (MTase), an essential enzyme for maintaining genomic stability. The sensor consists of a DNA probe, which contains up to three rolling circle amplification (RCA) primers sealed in one hairpin. MTase methylates the probe, which causes it to be digested by the restriction enzyme, releasing the primers. In the amplification step, the primers are elongated *via* RCA, producing a long ssDNA with multiple G-rich stretches. The latter can fold into GQs and induce NMM fluorescence in proportion to the amount of MTase present, with a detection limit of 0.0085 U/mL [61].

#### Studying enzyme activity and inhibition.

Sensitive, fast, easy, and cost-effective methods to study enzyme activity are highly desired, as enzymes are essential to many biological processes. Two NMM-GQ reporter systems were engineered to investigate activity and screen inhibitors for RNase H [62] and uracil-DNA glycosylase (UDG) [54].

RNase H catalyzes RNA hydrolysis in RNA-DNA duplexes and is important for DNA replication and repair. HIV reverse transcriptase — which is essential for retroviral DNA synthesis and subsequently host cell infection and viral replication — displays RNase H activity, making it of great interest for HIV/AIDS drug development. The biosensor for RNase H consists of a G-rich DNA-RNA hybrid and NMM, which displays low baseline fluorescence [62]. Once added to the system, RNase H digests the RNA, freeing the G-rich DNA to fold into a GQ that enhances NMM fluorescence by >10 fold. The authors demonstrate that NMM does not interfere with RNase H activity. This assay is fast (enzymatic activity can be detected within 5–10 min) and has a low detection limit of 0.2 U/mL [62]. Using a catalytic hairpin assembly signal amplification strategy, the detection limit was dramatically improved to 0.037 U/mL [63].

UDG catalyzes excision of uracil from DNA. The system to study UDG kinetics is a variation on the ssDNA biosensor presented earlier. It consists of the GQ-forming oligo P1 (which has G-rich portions on either side of a loop) hybridized with the oligo P2, which is complementary to the loop and contains four uracils. Hybridization of P1 and P2 prevents GQ formation, resulting in low fluorescence. When UDG is present, it digests the uracils in P2 and releases P1 to fold into a GQ, thereby resulting in a 16-fold increase in NMM fluorescence [54].

### Using NMM to discover new GQs and GQ ligands

Inspired by the Sen report, which suggested that NMM likely recognizes the GQ structure [10], the Johnson laboratory coupled NMM to a Sepharose resin using the carbodiimide method *via* the propionic acid side chains of NMM [64]. We later showed that the propionates play a minor role in NMM-GQ binding [2], so their modification should be inconsequential. Using the resin, Johnson’s lab demonstrated an excellent correlation between the predicted propensity of G-rich DNA to fold into GQ structures and the extent of the DNA binding to the resin. The methodology can be used to identify and isolate GQ DNA from *in vivo* sources illuminating genomic distribution of GQs.

Another method to detect GQs was developed by the Myong laboratory. It relies on the ability of NMM to quench the fluorescence of a 3′-Cy3 label upon close contact. Because of NMM’s selectivity, this methods worked well for parallel GQs [17]. To extend the assay, crystal violet (CV) was added to detect also antiparallel GQs [65]. The assay was validated by comparing its results to those from smFRET, which allow for direct quantification of parallel GQ, antiparallel GQ, and unfolded DNA. The NMM/CV assay is simple, reliable, quantitative, and can allow for high-throughput screening of genomic DNA. It is an alternative to smFRET and can complement the CD signatures of GQs which are currently used to infer specific GQ conformations. The Myong laboratory applied this assay to determine GQ topology as a function of loop composition and length [17], as well as to investigate the ability of G-rich DNA to fold in the context of dsDNA in both dilute and crowded conditions [65].

The Sugimoto laboratory developed a novel method to monitor co-transcriptional folding of RNA into GQs in real-time using NMM and the hydroxyethyl analogue of ThT, ThT-HE [66]. Both ligands selectively recognize the parallel GQ conformation that is usually adopted by RNA [82]. Crowding conditions (20 wt% PEG200) were used to mimic biological settings. The authors demonstrated that NMM serves merely as a reporter of GQ folding and does not affect the kinetics of the folding process. This method allows researchers to investigate the effect of a variety of intracellular factors on co- and post-transcriptional RNA folding in real time.

The Granzhan laboratory recently developed a high-throughput sensor array to identify DNA secondary structures with high accuracy and discover new DNA structural motifs [27]. The array includes 34 DNA sequences with representative topologies (ssDNA, dsDNA, parallel, antiparallel, and hybrid GQ DNA) along with 11 commercial dyes with documented affinity for DNA, including NMM. The authors demonstrated that NMM displays a selective fluorescence response to all GQs (the highest response in the presence of parallel GQs) but not to dsDNA or ssDNA [27], in agreement with other fluorescence enhancement studies [18, 19]. To simplify the array while maintaining its differential power, the 11 dyes were reduced to eight. This shortened list included NMM, which was deemed among the top three dyes in terms of its efficacy. The array can be continually trained using additional DNA sequences with well-characterized secondary structures to improve reliability of identification of DNA with known folds and to discover new DNA topologies.

The fluorescence indicator displacement (FID) assay is a widely used method to search for new GQ ligands [67, 68]. It relies on the ability of thiazol orange (TO) dye to fluoresce in the presence of GQs with different topologies. GQ binders will displace TO decreasing its fluorescence and the extent of the decrease could be used as a quantitative measure of ligand’s affinity for GQ. As demonstrated by the Bolton laboratory in their Mix and Measure assay, NMM can be used in a manner similar to TO. The assay contains 11 DNA sequences with representative GQ topologies (parallel, hybrid, and antiparallel) and three dyes: NMM and two carboxycyanines (DODC and DTDC) [20]. The use of multiple dyes, just like in Granzhan’s assay, yields redundant and complementary information, allowing for substantial improvement in the accuracy of ligand identification. The authors validated the assay using six known GQ ligands. As with FID, the Mix and Measure method is prone to false positives due to possible ligand-dye interactions, as well as false negatives if the ligand binding pocket is different than that of the dyes used in the assay.

In summary, the methods described in this section allow for a relatively simple, cost-efficient, and rapid identification of DNA secondary structures, discovery of novel DNA folds, and identification of new GQ ligands.

### Using NMM to study GQ DNA in biological contexts

**Fig 9.**
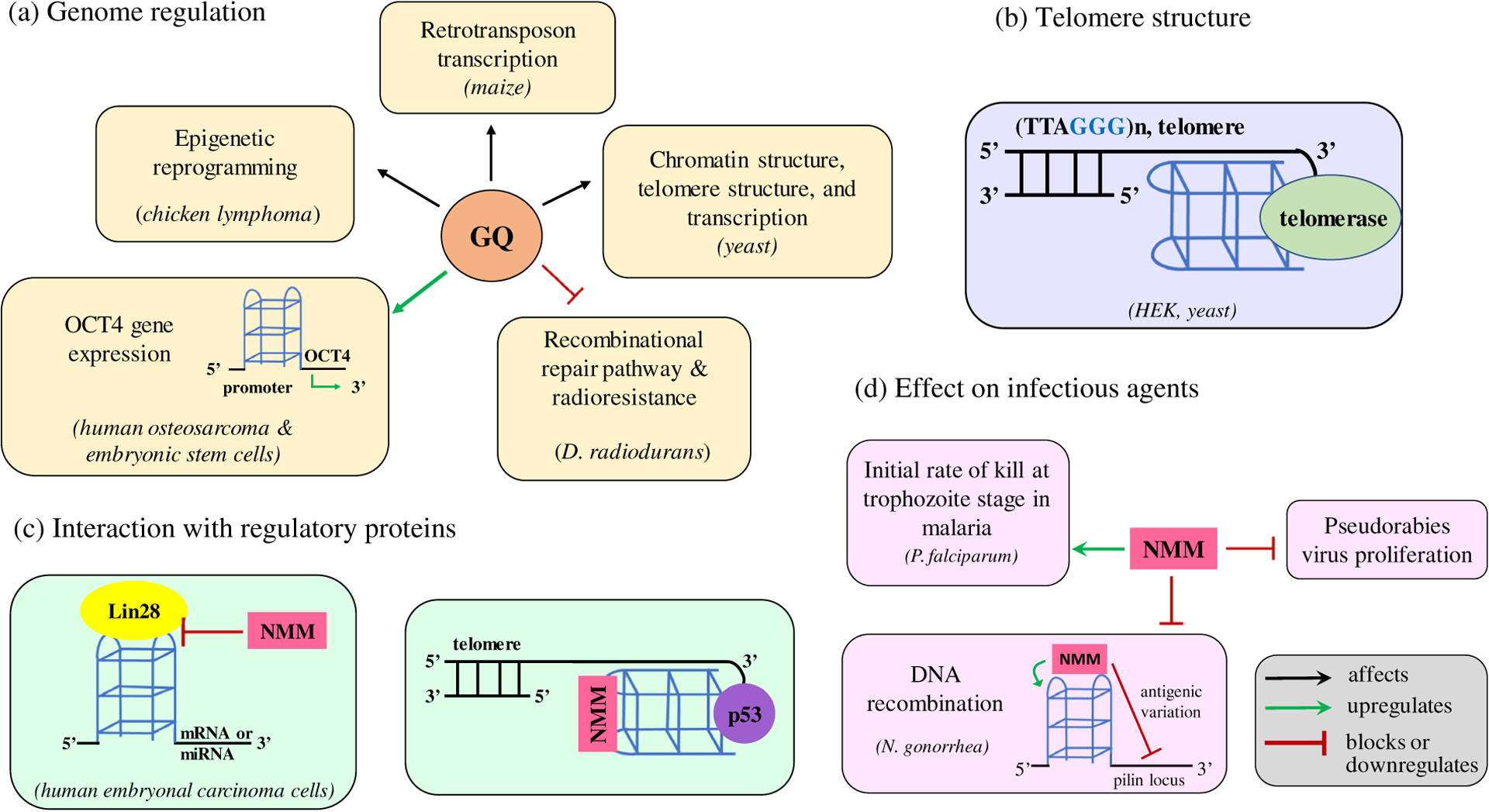
Applications of NMM to investigate the biological roles of GQ DNA. (a) Studies with NMM demonstrated that GQs are involved in genome regulation. (b) NMM revealed that parallel GQs form at human telomeres and can serve as rudimentary telomere caps. (c) NMM inhibits Lin28 binding to its RNA GQ substrates and promotes p53 binding to telomeric GQs. (d) NMM attenuates malaria and PRV pathogenicity. In the *N. gonorrhea* bacterium, NMM stabilizes a GQ upstream of the only expressed pilin locus to prevent antigenic variation

The ability of NMM to selectively recognize and stabilize GQ structures, along with its turn-on fluorescence in the presence of GQ DNA is particularly valuable for investigating the most crucial questions in the quadruplex field: to what extent do GQs form *in vivo*, and what are their roles within the biological processes they have been implicated in? Thus far, the effect of NMM has been studied in a variety of cultured cells: human kidney cells (HEK293T) [69], human embryonic stem cells (CCTL14) [70], human embryonal carcinoma cells (NCCIT) [66], human osteosarcoma cells (U2OS) [70], porcine kidney cells (PK-15) [71], and chicken lymphoma cells (DT40) [72]. The effects of NMM have also been studied *in vivo* in a variety of organisms including yeast (*Saccharomyces cerevisiae*) [73, 74], the malaria parasite (*Plasmodium falciparum*) [75], the gonorrhea bacterium (*Neisseria gonorrhoeae*) [76], extremophiles (*Deinococcus radiodurans* and *Deinococcus geothermalis*) [77], and maize (*Zea mays*) [78], as well as in the pseudorabies virus [71]. *In vitro* data strongly suggests that NMM selectively binds to and stabilizes parallel GQs (Fig. 3 and Fig. 5) even in the presence of high concentrations of competing dsDNA [7, 19]. This ability is also believed to be true *in vivo*, although it is possible that interactions with other biomolecules (*e.g.* ferrochelatase) might also contribute to NMM’s biological effects. Nonetheless, as NMM preferentially acts on regions of the genome with GQ-forming potential, it is likely that NMM has direct effects on GQs *in vivo*. NMM has made great contributions to the quadruplex field by providing substantial evidence that GQ-forming nucleic acids hold biological significance (Fig. 9).

#### Studying genome regulation by GQs.

Growing maize seedlings in NMM (16 μM for 3 days) differentially affected the transcription of various G-rich long terminal repeat retrotransposons (self-amplifying genetic elements) [78]. This study reveals that GQs may influence genome dynamics by regulating retrotransposon expression.

Treatment of exponentially growing yeast with NMM (8 μM) inhibited its growth rate by 25% and led to the preferential regulation of genes associated with chromatin structure, telomere structure, and transcription. NMM caused cells to accumulate in the S-phase, but had no effect on S-phase-regulated genes. Beyond cell cycle perturbation, NMM treatment led to upregulation of 9% of genes, including many promoters and open reading frames with high quadruplex forming potential. At the same time, 9% of genes were downregulated, including many involved in nucleolar functions such as rRNA processing and ribosome biogenesis, which may be impacted by rRNA GQ formation. Together, these results indicate that NMM-stabilized GQs affect yeast growth and gene expression [73].

Treating the radioresistant bacteria *D. radiodurans* with NMM (50 nM) caused radioresistance to decrease by ~60% (when exposed to 10 kGy gamma irradiation) [77]. It is remarkable that biological effects were observed at NMM concentrations below the average reported Kd (0.1–1 μM, Table 2). However, NMM binds some GQs with exceptionally high affinity (as observed for THM with a Kd of ~20 nM), and it is possible that NMM is concentrated within the cell. NMM may also act *via* targets other than GQs. The increase in radiation sensitivity was accompanied by the radiation dose-dependent downregulation of RecF recombinational repair pathway genes (*recA, recF, recO, recR,* and *recQ*), whose promoters have high GQ-forming potential. This work demonstrates that GQs contribute to radioresistance gene regulation, thereby establishing the connection between GQs and radioresistance for the first time and showing that GQ stability affects organismal function.

OCT4, a pluripotency gene highly expressed in both cancers and stem cells, has a conserved region near its transcription start site that is proposed to fold into a parallel GQ structure [70]. When OCT4 gene expression in human osteosarcoma and embryonic stem cells was interrupted by a single nucleotide polymorphism that destabilized the GQ, NMM treatment (30 μM for 12 h) rescued OCT4 gene expression. Meanwhile, NMM did not have a statistically significant effect on wild-type cells. Thus, NMM helped to demonstrate that GQ formation at the OCT4 promoter acts as a positive regulator of OCT4 gene expression and thereby identify the OCT4 locus as a potential target for GQ-based anticancer therapy [70].

Finally, treating chicken lymphoma cells with NMM (5 μM) impeded DNA replication at a G-rich locus (*BU-1*) and triggered local, heritable epigenetic changes that decreased gene expression at that locus. This proof-of-concept study demonstrates that stabilization of DNA secondary structures (*e.g.* GQs) is a viable approach to inducing locus-specific epigenetic reprogramming and presents a novel method for epigenetic therapy [72].

In summary, treatment of cells and living organisms with NMM suggests that GQs likely regulate gene expression, modulate the epigenetic landscape of the genome, and affect cell growth and radioresistance, among other biological functions.

#### Investigating telomere structure and capping.

Telomeres are repetitive regions found at the ends of chromosomes which serve to maintain genome integrity. Vertebrate telomeres generally include a ~100 nucleotide single-stranded 3′ overhang consisting of a G-rich repeat (*e.g.* TTAGGG in humans). In 2005, Paeschke *et al*. demonstrated telomeric GQ formation *in vivo* in the ciliate protozoan *Stylonychia lemnae* [79].

Treating human embryonic kidney cells with NMM increased the number of parallel GQs at human telomeres and the co-localization between GQs and telomerase, while it had no effect on cell cycle progression into mid-S phase [69]. Additionally, NMM treatment slightly rescued the growth of yeast with a mutant telomere-capping gene (*cdc13–1*) [74]. This result suggests that telomeric GQs serve as a rudimentary telomere cap when natural protein capping is compromised. Both studies provide evidence that GQs play important roles in telomeres.

#### Identifying GQs as targets for regulatory proteins.

NMM was instrumental in demonstrating that GQs can be recognized by regulatory proteins such as Lin28 [80] and p53 [81]. NMM was identified as the first small molecule inhibitor of Lin28, a conserved RNA-binding protein overexpressed in many cancers and linked to poor prognoses. In particular, Lin28 promotes translation by remodeling GQs within its mRNA substrates and also inhibits miRNA processing [80]. RNA GQs are uniformly parallel [82] (with one exception [83]), making them an excellent target for NMM. Treatment of human embryonal carcinoma cells with NMM (100 μM for 48 h) led to accumulation of mature miRNA, decreased levels of pluripotency proteins corresponding to Lin28 mRNA targets (*OCT4*, *HMGA1*, *CCNB1*, *CDK4*, and *Lin28A*), and decreased levels of two Lin28 mRNA targets (MYC and Lin28). NMM also inhibited self-renewal of cells and reduced their stem cell traits. Since NMM and Lin28 recognize the same RNA GQ features, NMM can inhibit Lin28 binding and thereby potentially prevent tumor progression in cancers.

The tumor suppressor protein p53 binds telomeric DNA in a structurally selective manner. Specifically, this binding interaction is enhanced in the presence of NMM/K^+^ but not Na^+^ (which typically stabilizes antiparallel GQs) or Li^+^ (which does not support GQ formation). Moreover, NMM had no effect on p53 binding to non-G-rich DNA. These findings suggest that p53 selectively recognizes parallel GQ structures [81].

In sum, these studies indicate that depending on the context, NMM can inhibit or enhance the binding of a regulatory protein to its GQ target. Their findings suggest that NMM can serve as either a competitive inhibitor or an allosteric activator, respectively. When NMM and a GQ-binding protein recognize the same GQ structural feature, competition for this binding site may lead to inhibition of protein binding. However, when a protein recognizes a different site on the GQ, NMM can promote protein binding by stabilizing the GQ structure. Using NMM as a selective probe for GQs, the two studies demonstrate that regulatory proteins can recognize GQ structures. In doing so, they provide further evidence for the roles of GQs in biological processes and also highlight GQ-protein interactions as potential targets for anticancer therapy.

#### Effect of NMM on infectious agents.

NMM has been identified as a potential drug against the malaria parasite [75], the pseudorabies virus [71], and antigenic variation in the gonorrhea bacterium [76]. These effects are directly linked to NMM’s ability to induce and stabilize GQ structures, thereby indicating that GQs are viable targets in infectious diseases.

A proof-of-concept study demonstrated that NMM (84 μM) displayed rapid cytocidal activity (*i.e.* rate of kill in trophozoite stage) in *P. falciparum* comparable to that of the common antimalarial drug chloroquine [75]. The biological effect of NMM is likely due to deregulation of expression of genes with high quadruplex-forming potential. Indeed NMM markedly suppressed expression of GQ-forming reporter genes. This study was the first to demonstrate the presence of GQs in malaria parasite nuclei and to establish GQs as antimalarial targets as well as GQ ligands as potential antimalarial agents.

Similarly, NMM (150 nM for 24 h) reduced the viral load (titer) of the pseudorabies virus (PRV) Ea strain in porcine kidney cells. It likely did so by stabilizing two-tetrad GQs, which were found to be predominantly parallel [71]. PRV is a swine herpesvirus that serves as a model system for studying herpesvirus biology but has been recently reported to cause endophthalmitis in humans [84].

Additionally, NMM was shown to inhibit DNA recombination in *N. gonorrheae*, the bacteria responsible for gonorrhea [76]. DNA recombination in *N. gonorrheae* leads to antigenic variation (Av), which allows the bacteria to escape the host’s immune response and prevents the development of a vaccine. One of the Av systems in *N. gonorrheae* involves pili — hair-like structures grown on the surface of many bacteria that allow them to attach to host cells. A G-rich sequence identified near the pilin locus was shown to fold into a GQ structure *in vitro*. Any GQ-disrupting mutations or treatments reduced pilin Av. Interestingly and seemingly contradictorily, NMM (at 0.38 μM, a concentration that does not alter bacterial growth) also reduced pilin Av and pilus phase in spite of its GQ-stabilizing ability. This result may be explained by the fact that to initiate recombination, a nick or break in DNA is required, and NMM was shown to prevent nicks on the G-rich strand [76]. Demonstrating involvement of GQs in Av and identifying NMM as a potential inhibitor of Av has great implications for both understanding and combating microbial pathogenesis.

Together, these studies indicate that NMM is capable of attenuating pathogenicity by selectively binding and stabilizing GQs, thereby establishing GQs as promising therapeutic targets against bacteria, viruses, and parasites. These findings are especially important in light of the growing resistance to current first-line therapeutic strategies against these pathogens.

### NMM as a photosensitizer in photodynamic therapy

Photodynamic therapy (PDT) refers to light-activated toxic effects produced by photosensitizers (*e.g.* porphyrins) on cancer cells. A 1998 study by the Solomon lab indicated that NMM may be a viable photosensitizer for PDT either on its own or in combination with PPIX [85]. Specifically, the authors suggest that porphyrins, known to accumulate in mitochondria, disrupt mitochondrial function by binding to mitochondrial benzodiazepine receptors (MBR), and that their affinities for MBR correlate with their photodynamic cell toxicity. NMM was the 7th best out of the 27 porphyrins studied, with 4 h of exposure at 0.35 μM followed by 60 s of irradiation reducing hamster lung cell survival to 37% after 7–14 days. Meanwhile, PPIX was the 3rd best, with the same effects being observed at 0.06 μM. To enhance the PDT effects, the amount of PPIX can be increased *via* addition of PPIX precursor, 5-aminolevulinic acid (ALA). Since NMM inhibits Fe^2+^ incorporation into PPIX [8], it helps maintain high PPIX levels, thereby further improving the PDT treatment.

A further proof-of-concept study for the use of NMM in PDT utilizes gold nanoparticles (AuNP) conjugated with a G-rich nucleolin aptamer AS1411 (also known as AGRO100) folded into a stable parallel GQ and decorated with NMM [86]. Nucleolin receptors are known to be overexpressed on cancer cells. Incubating HeLa cells with functionalized AuNPs (1 h at 10 nM) and subsequent irradiation for 30 min with white light led to a loss of >30% cell viability. Furthermore, these functionalized AuNP were successful in fluorescent imaging of HeLa cells, but not normal human kidney cells. Successful cell imaging results from NMM fluorescence induced by AS1411, while selectivity of imaging results from the specific interactions between AS1411 and nucleolin. Together, these findings highlight NMM as both a viable photosensitizer for PDT and an essential component of a new fluorescent probe for cancer cells.

### Non-GQ based applications of NMM

After the original discovery of NMM’s ability to inhibit Fe^2+^ insertion into PPIX, NMM was used to investigate heme biosynthetic pathways in plants. In the early 80s, Beale and Foley showed that treatment of blue-green algae, *Euglena gracilis*, with NMM (4 μM, 6 h) increased the activity of ALA synthase and decreased heme levels without affecting chlorophyll (Chl) production, which indicated that ALA is not a precursor for Chl [87]. Treating cyanobacteria, *Cyanidium caldarium,* with NMM (3 μM, 72 h) indicated that heme was a metabolic precursor to a light-harvesting pigment phycocyanin [88]. Finally, NMM (100–200 μM, 24 h) inhibited production of phytochrome, another light-harvesting pigment in plants, suggesting that phytochrome is an end product in the Fe^2+^ branch of the biosynthetic pathway in which PPIX and heme are intermediates [89]. In all these studies Chl production was not affected, suggesting that NMM does not inhibit Mg^2+^ insertion into PPIX.

Another application of NMM that does not rely on its GQ-binding ability concerns fibrils formed by oligomerization of the 40-residue amyloid-β peptide (Aβ40). Amyloid fibrils may act as neurotoxins and are involved in Alzheimer’s disease pathogenesis. NMM fluorescence was found to increase in the presence of fibrils but not the Aβ40 monomer [90]. Consequently, NMM could be used as an amyloid dye complementary to or in place of ThT, a common amyloid dye. While binding of NMM and ThT to Aβ40 is comparable (Ka ~5.0 × 10^5^ M^−1^), NMM is far superior to ThT due to its resistance to photobleaching and large Stokes shifts (210 nm). Its red emission allows detection of Aβ40 in the presence of intrinsically fluorescent cellular components as well as green fluorescent probes. Staining of Aβ40 fibrils with NMM (3–5 μM for 10 min) has been reported in both *E. coli* culture and rat pheochromocytoma cells (PC12) [90]. It therefore comes as a surprise that NMM has not yet been used to stain cells for GQs. Furthermore, NMM (10 μM for 7 days) was also shown to inhibit Aβ40 aggregation *in situ* by prolonging the nucleation phase and slowing the elongation phase. In sum, NMM can be used not only to probe for Aβ40 aggregates to monitor Alzheimer’s disease progression, but also to develop a small molecule inhibitor of fibril formation that can serve as an Alzheimer’s therapeutic.

## CONCLUSIONS

NMM is an excellent GQ binder whose greatest utility comes from its selective recognition of parallel GQ DNA, light-switch fluorescence properties, and water solubility. The importance of drug selectivity, especially those used for cancers, cannot be overstated, and NMM is superior in its selectivity to any other reported GQ binder. While it is possible that GQs formed in different regions of the genome work in concert, it is more likely they act independently, bringing about both positive and negative changes. Thus, small molecule ligands that exhibit broad GQ binding might induce a combination of desirable and undesirable changes. Meanwhile, NMM, with its unprecedented preference for parallel GQ structures, provides an excellent platform for investigating the structural and chemical requirements for highly selective GQ binders. Furthermore, NMM’s interaction with GQs has been used to develop biosensors for many different molecules, as well as to investigate the roles of GQs in a variety of biological processes. However, we should not forget that NMM can induce GQ structural rearrangement, so the results of NMM treatments in cells or *in vivo* must be interpreted with great care and consideration. In addition, the non-GQ-based effects of NMM, such as inhibition of ferrochelatase function, should also be considered. Open questions remain about NMM’s bioavailability, cytotoxicity, *in vivo* half-life, and degradation products, as well as its viability as a fluorescent GQ probe for cell staining. Moreover, the relationship between NMM’s binding affinity and the structure of its binding partner remains to be determined. These topics will require further investigation, and some of this work is currently underway in our laboratory.

## Figures and Tables

**Table 1.** DNA sequences mentioned in this review; stretches of guanines and cytosines are highlighted in red and blue, respectively

Name	Sequence 5′ to 3′	#nt	Secondary structure type/notes
**Tel22**	AGGGTTAGGGTTAGGGTTAGGG	22	Hybrid GQ in K^+^
**G**_**4**_ **T**_**4**_ **G**_**4**_	GGGGTTTTGGGG	12	Antiparallel GQ
**THM**	GGGTTGGGTTGGGTTGGG	18	Parallel GQ in K^+^
**VEGF**	GGGAGGGTTGGGGTGGG	17	Parallel GQ dimer and monomer
**26TelG4**	AGGGGTTAGGGGTTAGGGGTTAGGGG	26	Antiparallel GQ in Na^+^
**G4TERT**	AGGGGAGGGGCTGGGAGGGC	20	Parallel GQ
**TBA**	GGTTGGTGTGGTTGG	15	Antiparallel GQ
**cMyc**	TGAGGGTGGGTAGGGTGGGTAA	22	Parallel GQ
**cMyc**	TGAGGGTGGGGAGGGTGGGGAA	22	Used for split ssDNA sensor
**cKit1**	GGGAGGGCGCTGGGAGGAGGG	21	Parallel/hybrid GQ
**cKit2**	GGGCGGGCGCGAGGGAGGGG	20	Parallel GQ
**Bcl-2**	GGGCGCGGGAGGGAATTGGCGGGG	24	Parallel/hybrid GQ
**24GT**	GGGTTTTGGGTTTTGGGTTTTGGG	24	Antiparallel GQ
**KRAS-21R**	AGGGCGGTGTGGGAAGAGGGA	21	Parallel GQ
**AGRO100 or AS1411**	GGTGGTGGTGGTTGTGGTGGTGGTGG	26	Nucleolin aptamer that forms a parallel GQ
**PS2.M**	GTGGGTAGGGCGGGTTGG	18	GQ
**Sugimoto aptamer**	GTGGGTTGGGTGGGTTGG	18	GQ
**OCT4-WT**	agtGGGTGGGACTGGGGAGGGagagaggggttgagtagtccct	43	Parallel GQ; capital letters represent GQ forming region
**OCT4-SM**	agtGGGTGGGACTGGGGAGAGAGAGAGGGGttgagtagtccct	43	Parallel GQ; capital letters represent GQ forming region
**APS-WT**	AATGGGTTTGGGTTTGGGTTTGGGTAA	27	Antiparallel/hybrid GQ
**F21D**	FAM-GGGTTAGGGTTAGGGTTAGGG-Dabcyl	21	Human telomeric DNA used in FRET
**i-cMyc**	TTACCCACCCTACCCACCCTCA	22	i-motif
**ds26**	CAATCGGATCGAATTCGATCCGATTG	26	dsDNA FRET control

**Table 2.** Summary of binding affinity, Ka, of NMM for GQ DNA

Name	GQ conformation	Methods for measuring binding	Buffer**	[K^+^], mM	Binding constant (Ka)^#^, × 10^6^ M^−1^	Stoichiometry GQ:NMM	Reference
**THM**	Parallel, dimer	UV-vis titration FL titration ITC	5K	5	30 ± 20 50 ± 20 70 ± 20	1:1	Unpublished
**cMyc**	Parallel	UV-vis titration FL titration	BPEK	100	19 ± 6 7.4 ± 2	1:1	Unpublished
		Cy3 quenching	100K	100	~10		Tippana *et al.* [17]
**G**_**4**_ **T**_**4**_ **G**_**4**_	Antiparallel	FL titration	5K	5	12.6 ± 0.7	1:1	Sabharwal *et al.* [19]
**VEGF**	Parallel, dimer	UV-vis titration	5K	5	10. ± 2	1:1	Unpublished
**VEGF** *	Parallel monomer	UV-vis titration	5K	5	2.1 ± 0.5	2:1	Unpublished
**G4TERT**	Parallel, dimer	UV-vis titration	5K	5	1.7 ± 0.7	1:1	Unpublished
**G4TERT** *	Parallel, monomer	UV-vis titration	5K	5	6.1 ± 0.8	1:1	Unpublished
**Various GQ**	50–100% Parallel 0–50% Parallel 100% antiparallel and ssDNA	Cy3 quenching	100K	100	1–10 0.1–0.01 <0.01	—	Tippana *et al.* [17]
**T**_**4**_ **G**_**4**_ **T**_**4**_	Parallel	FL titration	TB	10	1.4 ± 0.2	1:1	Sabharwal *et al.* [19]
**T**_**8**_ **G**_**8**_ **T**_**8**_	Parallel	FL titration	TB	10	1.7 ± 0.2	1:1	Sabharwal *et al.* [19]
**Tel22**	Hybrid	UV-vis titration CD titration	5K	5	0.1 ± 0.03 0.07 ± 0.01	1:1	Nicoludis *et al.* [7]
**Sen aptamers**	Likely GQ	Affinity chromatography	SB	25	1.3–2.5	—	Li *et al.* [10]
**Sugimoto aptamer**	GQ	FL titration	TGK	5	1.4	—	Yang *et al.* [12]
**ZnP1.2 aptamer**	Likely parallel GQ	FL titration	SELEX	60 Na^+^	0.74	3:2	Li W *et al.* [14]
**Ten Yang aptamers**	GQ or hairpin	FL titration	TGK	5	0.8–1.1 0.2–0.3 < 0.05	—	Yang *et al.* [12]
**G**_**4**_ **T**_**4**_ **G**_**4**_	Antiparallel	FL titration	50Na	50 Na^+^	Nonspecific binding	—	Sabharwal *et al.* [19]
**26TelG4**	Antiparallel	FL titration	50Na	50 Na^+^	Nonspecific binding	—	Sabharwal *et al.* [19]

* Expanded variant designed to avoid dimer formation.

** Buffer composition.

5K: 10 mM lithium cacodylate 7.2, 95 mM LiCl, and 5 mM KCl.

TB: 50 mM Tris-borate 8.3, 1 mM MgCl2, and 10 mM KCl.

BPEK: 6 mM Na_2_HPO_4_, 2 mM NaH_2_PO_4_ 7.1, 1 mM Na_2_EDTA, and 100 mM KCl.

100K: 10 mM Tris–HCl pH 7.5, and 100 mM KCl.

TGK: 25 mM Tris 8.3, 192 mM glycine, and 5 mM KH_2_PO_4_.

SB: 100 mM Tris-acetate 7.4, 200 mM NaOAc, 25 mM KOAc, 10 mM Mg(Oac)2, 0.5% Triton X-100, and 5% DMSO.

50Na: 10 mM lithium cacodylate 7.2, 50 mM LiCl, and 50 mM KCl.

SELEX: 20 mM HEPES, pH 7.4, and 60 mM NaCl.

# Ka could be easily converted to Kd (widely used in biology) by the following relationship: Kd = 1/Ka.
